# Fitting item response unfolding models to Likert-scale data using mirt in R

**DOI:** 10.1371/journal.pone.0196292

**Published:** 2018-05-03

**Authors:** Chen-Wei Liu, R. Philip Chalmers

**Affiliations:** 1 Faculty of Education, the Chinese University of Hong Kong, Hong Kong, Hong Kong; 2 Quantitative Methodology, University of Georgia, Athens, United States of America; Leibniz Institute for Educational Trajectory, GERMANY

## Abstract

While a large family of unfolding models for Likert-scale response data have been developed for decades, very few applications of these models have been witnessed in practice. There may be several reasons why these have not appeared more widely in published research, however one obvious limitation appears to be the absence of suitable software for model estimation. In this article, the authors demonstrate how the **mirt** package can be adopted to estimate parameters from various unidimensional and multidimensional unfolding models. To concretely demonstrate the concepts and recommendations, a tutorial and examples of **R** syntax are provided for practical guidelines. Finally, the performance of **mirt** is evaluated via parameter-recovery simulation studies to demonstrate its potential effectiveness. The authors argue that, armed with the **mirt** package, applying unfolding models to Likert-scale data is now not only possible but can be estimated to real-datasets with little difficulty.

## Introduction

In item response theory (IRT) modeling for categorical data [1], two cognitive processes are typically modeled: the cumulative process, and the unfolding process. The former process postulates that the probability of responding to higher rank-ordered categorical response stimuli can be understood as a monotonic function that rises as the intensity of the person underlying trait increases (termed the cumulative process), while the latter process assumes the probability depends on the proximity between person and item location [2–4]. The cumulative process is commonly assumed for scholastic performance on science, mathematics, or other literacy tests. The partial credit model [5], for example, is one of the IRT models (also commonly referred to as dominance models) frequently used for polytomous items with a cumulative process, and is expressed as follows on a logit scale:

$$\log({\mathrm{Pr}}_{nik}/{\mathrm{Pr}}_{ni(k-1)})=\gamma_{n}-\kappa_{i}-\tau_{ik}.$$ (1)

In the above equation, Pr_*nik*_ and Pr_*ni*(*k*-1)_ are short for the item response function of scoring *k* and *k*– 1 on item *i* for person *n*, γ_*n*_ is the ability of person *n*, κ_*i*_ is the difficulty of item *i*,τ_*ik*_ (*k* = 0, …, *C*) is the *k*th threshold parameter of item *i*, which indicates the intersection of two adjacent item category functions, and *C* is the number of categories minus one. As a special case, the partial credit model encompasses the Rasch model [6] when the response data are binary.

### Unfolding models

The unfolding process postulates that the closer the distance between an individual’s latent trait and item location the higher the probability of endorsement to a given response category. In these IRT models, the probability reaches its peak when the individual’s trait is equal to the item location. The unfolding process has attracted great interests in constructs of personality, attitudes, job performance, vocational interests, leadership, emotion measurements, and so forth [7–13]. The unfolding model has also seen a wide variety of applications in that it has been applied to computerized adaptive testing [14, 15], response styles [16], computerized classification testing [17], multilevel data analysis [18], multidimensional latent scaling [19, 20], and random threshold modeling [15].

In social behavior surveys, [21] discussed two types of measurement stimuli—a direct-response item stimuli, such as Likert-type items (e.g., strongly disagree, disagree, agree, strongly agree), and a comparative-response stimuli (e.g., pairwise preference). For these data, the underlying process of respondents may be explained by either the cumulative or unfolding process, depending on the nature of the response stimuli administered. The resulting combinations of processes and stimuli will therefore be one of the following two-by-two arrays: [cumulative process and direct response], [cumulative process and comparative response], [unfolding process and direct response], and [unfolding process and comparative response]. In the present study, the focus is on the third combination.

For the combination of unfolding process and direct response, asking individuals to express their level of agreement with an attitude statement, for example, provides information as to whether they agree with the item to the extent that it is close to their location on the latent trait continuum. On the contrary, a negative response to this stimulus may result because the respondent may disagree with the item statement from either a negative *or* positive perspective. For example, consider the Likert-scale item, “I think capital punishment is necessary but I wish it were not” [3]. Participants with more positive attitudes towards capital punishment tend to disagree with this statement because they believe that capital punishment is very necessary (positive), whereas participants with more negative attitudes towards capital punishment are also likely to disagree because they believe capital punishment is very unnecessary (negative). Hence, there are two possible latent responses—“disagree from below” and “disagree from above”—associated with the single observed response of “disagree.” As such, a U-shape item response function is generally more appropriate to illustrate the two disagreements, where the probability of agreement typically follows a single-peaked item response function.

Regarding the types of unfolding models to fit to empirical data, there are two general statistical modeling strategies—the first is a parametric approach, and the second is a nonparametric approach [22]. The nonparametric approach does not assume any specific form of the item response function, and only considers the proximity between person and item. A typical nonparametric approach is the Coombs scaling [2], whose purpose is to map the proximity between persons and items into a lower dimensional representation for visual illustration (cf. **smacof** package). Unfortunately, Coombs scaling is a non-probabilistic type of unfolding approach, and is mainly used for visual presentation of data in two or three dimensions. In contrast, parametric unfolding models aim to scale persons and items under a probabilistic framework, which are typically more useful for subsequent applications [23]. Thus, compared to the Coombs scaling, the parametric unfolding models are more advantageous for model-data fit assessment, model comparison, prediction of persons’ current and future ratings, assessment of differential item functioning, applications to computerized adaptive/classification testing, among others. Therefore, in this study we chose to focus only on parametric unfolding models and how to recover the population generating parameters for various unfolding models of interest.

### Available estimation software

The most commonly used IRT unfolding model is the generalized graded unfolding model [GGUM; 24]. In part, the popularity of the GGUM may be contributed to its distribution of the freeware software package GGUM2004 [25], which allows many variations of the GGUM model to be estimated via maximum-likelihood and maximum a posteriori methods. However, the item characteristic curve (ICC) kernel of the GGUM cannot be changed; hence, this limits the capability of fitting unfolding data outside the form supported by the GGUM. Additionally, while the τ coefficients modeled generally represent the location of intersection between adjacent ICCs in the partial credit model (see below for notation), such interpretation disappears in the GGUM parameterization [24]. To circumvent this limitation, the authors demonstrate how to employ an alternative class of unfolding models proposed by Luo [26], which can provide more flexible ICCs and boast more explicit interpretations of the threshold parameters.

To date, no general-purpose estimation software has been made available specifically for unfolding models, except perhaps the general purpose Markov chain Monte Carlo (MCMC) sampling software (e.g., JAGS [27]) and RUMMFOLD software [28]. However, conducting MCMC is practically time-consuming and demands relevant expertise on Bayesian inference (e.g., specifying proper prior distribution, assessing convergence, etc.) [15, 20, 29], while the program RUMMFOLD is currently restricted to one specific unidimensional unfolding model for binary response data. Various sorts of popular IRT software for dominance models, such as ConQuest [30], Winsteps [31], BILOG-MG [32], IRTPRO [33], and so forth, have been developed for calibrating the parameters of cumulative IRT models. However, despite their popularity, none of these software packages are currently capable of estimating IRT unfolding models. As an alternative to these commercial IRT programs, the authors propose using the open-source **mirt** [34] package in the R environment for parameter estimation of the unfolding models. **mirt** has been widely used in educational measurement [35], personality assessment [36, 37], and IRT modeling [38], for cumulative IRT models, yet few authors are aware that **mirt** can be used to create real-world, fully customized IRT models; including, but not limited to, a wide variety of developed unfolding models.

According to the user software manual, GGUM2004 only allows for maximum (1) 2,000 subjects, (2) 100 items, (3) 10 categories of an item, (4) 50 quadrature points for marginal maximum likelihood estimation with expectation-maximization (MML-EM) algorithm [39], (5) prior standard normal distribution for latent trait [25], (6) only expected a posteriori (EAP) estimates are available, and (7) requires unidimensionality. In contrast, **mirt** by default is free from all these practically limiting restrictions. Although **mirt** adopts a normal distribution for latent traits by default, for instance, it also allows for estimating the mean and variance of the distribution (so long as the model is well identified). GGUM2004, on the other hand, assumes the standard normal distribution for eight models available in the GGUM2004, which often can lead to over-constrained estimation of a selection of GGUMs.

To demonstrate the usefulness of the **mirt** package in fitting unfolding models, the remainder of this article is organized as follows. First, a class of unidimensional unfolding models for Likert-scale items is introduced, which includes eight models of GGUM2004 and Luo’s general unfolding models [26]. Second, a class of Luo’s multidimensional unfolding models for Likert-scale items [40] is introduced. Following this introduction, a series of Monte Carlo simulation studies are conducted to investigate the parameter recovery of the various unfolding models using **mirt**, including (1) direct comparisons between **mirt** and GGUM2004, (2) parameter recovery of Luo’s unidimensional models, and (3) parameter recovery of Luo’s multidimensional models. Results are presented in each respective study, and concluding remarks are given in the final sections.

## Unidimensional unfolding model for Likert Scale Data

### Unfolding models of GGUM2004

In the following simulation studies, eight models estimable by GGUM2004 (version 1.1) were adopted [25]. We begin by discussing the sixth model in the command options because of its generality. This model is known as the generalized multiple unit unfolding model (denoted UM6 for short), given by

$$\mathrm{Pr}(z)=\frac{\exp\left\{\alpha_{i}\left[z\left(\theta_{n}-\delta_{i}\right)+z\left(M-z\right)\lambda_{i}\right]\right\}+\exp\left\{\alpha_{i}\left[\left(M-z\right)\left(\theta_{n}-\delta_{i}\right)+z\left(M-z\right)\lambda_{i}\right]\right\}}{\sum_{w=0}^{C}\exp\left\{\alpha_{i}\left[w\left(\theta_{n}-\delta_{i}\right)+w\left(M-w\right)\lambda_{i}\right]\right\}+\exp\left\{\alpha_{i}\left[\left(M-w\right)\left(\theta_{n}-\delta_{i}\right)+w\left(M-w\right)\lambda_{i}\right]\right\}},$$ (2)

where *z* is the observed value of categorical random variable *Z*_*nik*_, *M* = 2*C* + 1, *C* is number of categories minus one, and the λ_*i*_ is the unit threshold for item *i* [25]. In this model, a total of 3*I* item parameters (i.e., α_*i*_, δ_*i*_, and λ_*i*_) are to be estimated, where *I* is the number of items. When α_*i*_ = 1, UM6 reduces to the multiple unit model (denoted UM2). When λ_*i*_ = λ, UM6 reduces to the generalized constant unit model (denoted UM5). When α_*i*_ = 1 and λ_*i*_ = λ, UM6 reduces to the constant unit unfolding model (denoted UM1).

The GGUM itself [24], denoted UM8, is given by

$$\mathrm{Pr}(z)=\frac{\exp\left\{\alpha_{i}\left[z\left(\theta_{n}-\delta_{i}\right)-\sum_{k=0}^{z}\tau_{ik}\right]\right\}+\exp\left\{\alpha_{i}\left[\left(M-z\right)\left(\theta_{n}-\delta_{i}\right)-\sum_{k=0}^{z}\tau_{ik}\right]\right\}}{\sum_{w=0}^{C}\exp\left\{\alpha_{i}\left[w\left(\theta_{n}-\delta_{i}\right)-\sum_{k=0}^{w}\tau_{ik}\right]\right\}+\exp\left\{\alpha_{i}\left[\left(M-w\right)\left(\theta_{n}-\delta_{i}\right)-\sum_{k=0}^{w}\tau_{ik}\right]\right\}},$$ (3)

where τ_*ik*_ is the threshold *k* of item *i*. A total of 2*I* + *CI* item parameters (i.e., α_*i*_, δ_*i*_, and τ_*ik*_) are to be estimated. When α_*i*_ = 1, UM8 reduces to the partial credit unfolding model (denoted UM4). When τ_*ik*_ = τ_*k*_, UM8 reduces to the generalized rating scale unfolding model (denoted UM7). Finally, when α_*i*_ = 1 and τ_*ik*_ = τ_*k*_, UM8 reduces to the graded unfolding model [41], denoted UM3. Note that the GGUM2004 constrains the latent trait variance parameter σ^2^ = 1 for UM1-UM4, which is not always necessary. To demonstrate this, σ^2^ was freely estimated by **mirt** for UM1-UM4.

Of the models mentioned above, the UM3 (graded unfolding model) and UM8 (GGUM) have attracted the most attention in the literature. Also, in order to make the narrative of this article coherent and reduce excessive acronyms, the authors only focus on UM3 and UM8 for illustration purposes in the example sections.

### Luo’s unfolding models

In 2001, Luo introduced a general form for unidimensional unfolding models, which is expressed as follows. Let *Z*_*ni*_ ϵ (0, 1, …, *C*) be the categorical score to item *i* for person *n*, whose category probability Pr(*Z*_*ni*_) is equal to a product of *C* successive binary operational probabilities, where *Y*_*nik*_ ϵ (0, 1) and *k* = 1, 2, …, *C*. The probability of polytomous response *Z*_*nik*_, given the person and item parameters, is

$$\mathrm{Pr}(Z_{nik}=z)=\frac{\prod_{k=1}^{C}P_{nik}^{U_{zk}}Q_{nik}^{1-U_{zk}}}{\sum_{w=0}^{C}\prod_{k=1}^{C}P_{nik}^{U_{wk}}Q_{nik}^{1-U_{wk}}},$$ (4)

where the dummy variable *U*_*zk*_ = 1 if *z* ≥ *k* and *U*_*zk*_ = 0 otherwise, and Q_*nik*_ = 1 –P_*nik*_. As well, *U*_*wk*_ = 1 if *w* ≥ *k*, and *U*_*wk*_ = 0 otherwise. P_*nik*_ is defined as

$$P_{nik}=\mathrm{Pr}(Y_{nik}=1)=\frac{\psi_{k}(\rho_{k})}{\psi_{k}\left[\alpha_{i}(\theta_{n}-\delta_{i})\right]+\psi_{k}(\rho_{k})},$$ (5)

where α_*i*_ is the discrimination parameter of item *i*, θ_*n*_ is the latent trait of person *n*, δ_*n*_ is the location of item *i*, andρ_*k*_ is the threshold parameter [26]. In the above equation, ψ(·) represents the operational function that must satisfy the following properties to form a valid unfolding response function [26, 42]: (1) non-negativity: ψ(*x*)≥0 for any real *x*, (2) monotonicity in the positive domain: ψ(*x*)> ψ(*y*) for any *x* > *y* > 0, and (3) symmetry of the function: ψ(*x*) = ψ(-*x*)for any real *x*. The *x* can be, for example, α_*i*_ρ_*k*_ or α_*i*_(θ_*n*_—δ_*i*_), as in Eq (5).

This model formula has two main advantages over the GGUM. The first is the flexibility of the operational function, and the second is that the threshold parameter, ρ, has direct interpretation—that is, the crossing point between curves of adjacent categories is the threshold location from the reference point, which incidentally corresponds to the .5 probability of endorsement. As well, different operational functions can lead to different shapes of item characteristic curves. In the interested of space, the authors only consider five operational functions.

The first operational function studied with Luo’s model is

ψ(x)=exp(|x|), (6)

which is called an absolute logistic model (ALM) [43], where *x* is a real number. The resulting probability density is similar to Laplace distribution but, in contrast, the ALM contains explicit threshold parameters. The prominent part of ALM is that the location of the threshold corresponds to the two peaks of the information function for binary responses [43]. That property may be useful to easily construct a customized item pool for computerized adaptive testing.

The second operational function we consider is

$$\psi \left(x\right)=\exp\left(x^{2}\right),$$ (7)

which is the simple squared logistic model (SSLM) [3], while the third model studied is

$$\psi \left(x\right)=x^{2},$$ (8)

which is called the Parallellogram Analysis model [PARELLA; 44]. The ICC of PARELLA model has an endorsement of probability of 1 if θ = δ. The fourth model studied herein is hyperbolic cosine model [HCM; 45, 46] whose operational function is

ψ(x)=cosh(x). (9)

Finally, the fifth operational function studied is

$$\psi \left(x_{k}\right)=\frac{\cosh\left[\left(\frac{2C+1}{2}+1-k\right)x\right]}{\cosh\left[\left(\frac{2C+1}{2}-k\right)x\right]},$$ (10)

which is called graded unfolding model [GUM; 26]. For these unfolding models, the item characteristic curves (ICCs) and Fisher information function of θ are given in S1 Fig, where the definition of the Fisher information of θ is given by

$$I\left(\theta \right)=E\left\{{\left[\frac{\partial }{\partial \theta }\log\;\mathrm{Pr}\left(Z|\theta \right)\right]}^{2}\right\}=\sum_{z=0}^{C}{\left[\frac{\partial }{\partial \theta }\log\;\mathrm{Pr}\left(z|\theta \right)\right]}^{2}\mathrm{Pr}\left(z|\theta \right).$$ (11)

Interested readers can refer to Luo and Andrich [43] for the properties of ICC and Fisher information.

To further demonstrate the difference between the unfolding model and dominance model, the ICCs of partial credit model [5] and various unfolding models are depicted in S1 Fig. When θ = 0 and δ = 0, it is obvious that the unfolding models reach the peak of probability of a positive response, whereas the PCM reaches the .5 probability. The probability of the PCM monotonically increases as the values of θ increases, irrespective to δ. In contrast, the ICC of unfolding models depends on the relative distance between θ and δ, which reflects the proximity concept of an unfolding process [2].

## Multidimensional unfolding model for Likert Scale Data

In addition to the unidimensional models presented in the previous section, Luo [40] discussed a class of multidimensional unfolding model (MUM) which replace the θ_*n*_−δ_*i*_ component by some distance between ||**θ**_*n*_−**δ**_*i*_ ||. This can be expressed as

$$P(Y_{nik}=1|\theta_{n},\delta_{i},\rho_{dk})=\frac{\psi_{k}(\rho_{dk})}{\psi_{k}(\left\|\theta_{n}-\delta_{i}\right\|)+\psi_{k}(\rho_{dk})},$$ (12)

where **θ**_*n*_ = (θ_*n*1_, θ_*n*2_, …, θ_*nD*_) and **δ**_*i*_ = (δ_*i*1_, δ_*i*2_, …, δ_*iD*_) are vectors with *D* dimension coordinates. There are various candidate measures of the distance possible for these models. One simple approach is the Euclidean distance between **θ**_*n*_ and **δ**_*i*_ in the *D*-dimension space

$$\left\|\theta_{n}-\delta_{i}\right\|=\sqrt{\sum_{d=1}^{D}{\left[\alpha_{id}\left(\theta_{nd}-\delta_{id}\right)\right]}^{2}},$$ (13)

where the α_*id*_ is a discrimination parameter of item *i* and dimension *d*. When α_1_ = 1 and α_*d* ≠ 1_ = 0, the distance becomes θ_*n*_−δ_*i*_; thus, the MUM reduces to the UUM.

The MUM has several interesting properties. First, the model preserves proximity—the shorter distance the θ_*d*_−δ_*d*_, the higher probability of endorsement. In contrast to other unfolding models, this property is often not present [19, 20]. For illustration, the ICCs and Fisher information function of two-dimensional HCM are given in Supporting Information (see S3, S4 and S5 Figs). Second, different dimensions have different respective item locations, δ_*d*_, which represent the ideal item location on each *d*th dimension. Third, **α** can be used to specify which dimension an item measures; for instance, **α** = [α_1_, 0, α_3_] indicates only the first and third dimensions are measured within a given item.

The δ_*id*_ is the *i*th unobserved item location on *d*th dimension, which will increase as the number of dimensions increases. This model is useful for exploratory data analyses, in a manner similar to multidimensional scaling and exploratory factor analysis, in that it aims to discover a low-dimensional representation embedded in the high-dimensional space. However, such models will be over-parameterized for confirmatory modeling purposes. Typically, it is assumed that there is only one ideal item location, δ_*id*_ = δ_*i*_, for within-item multidimensional IRT models [47].

Additionally, the MUM has an additional threshold parameter for binary scoring, as well as for polytomous cases. Therefore, estimating all of the parameters in MUM may be demanding given the amount of data required to achieve sufficient stability and precision. The MUM is over-parameterized and imposing constraints is necessary for sufficient identification. Another approach used for educational data is to impose a design matrix by test developers or subject matter experts [48–51], where each item may only measure one or a few dimensions. With limited space, the authors focus on the between-item design, whereby each item solely measures one dimension [47].

To estimate the MUM there are three essential constraints that must be considered—(1) location, (2) scale, and (3) rotation [52]. A multivariate normal distribution is employed to deal with the first two indeterminacies; that is, the means of **θ** are set to zero and the variance-covariance matrix is a (potentially non-diagonal) symmetric matrix whose diagonal elements are ones. The rotational indeterminacy means that the axes could switch between dimensions during the estimation process. Minimum constraints are imposed by setting the α_*id*_ = 0 when *d* > *I* (i.e., *D*(*D*– 1)/2 zeros) [52] and δ_*id*_ = δ_*i*_. However, these minimum constraints do not necessarily stabilize the estimation in practical analyses [48–50, 53]. For cumulative multidimensional IRT models, at least two or three items measuring a single dimension are recommended for the compensatory model [53], and at least six items are suggested for the noncompensatory model [48–51].

## Marginal maximum likelihood with the expectation-maximization algorithm

This section provides a brief overview of the marginal maximum likelihood (MML) estimation criteria utilized by both the **mirt** and GGUM2004 software packages. For any defined IRT model, the logarithm of the marginal likelihood function given the response patterns **X** is

$$\log L\left(\xi ;X\right)=\sum_{s=1}^{S}\log\left[\int f\left(\theta \right)\prod_{i=1}^{I}\mathrm{Pr}{\left(Z_{i}=x_{si}|\theta ,\xi_{i}\right)}^{\chi \left(x_{si}\right)}d\theta \right],$$ (14)

where the **ξ** is the collection of all item parameters, *S* is the number of response patterns, *I* is test length, *C* is category length minus one, *f*(**θ**) is probability density function of the latent traits (typically assumed to be a multivariate normal distribution with mean **μ** and variance-covariance matrix **Σ)**, and the χ(*x*_*si*_) is the data indicator function where χ(*x*_*si*_) = 1if *Z*_*i*_ = *x*_*si*_ and χ(*x*_*si*_) = 0 otherwise. To locate the item parameter estimates by maximum likelihood, one has to find the values that can set the first-order derivatives of the log-likelihood function with respect to the parameters equal to zero. Unfortunately, however, solving the MML criteria directly is largely limited to shorter tests because the integrals run across all *I* items. To avoid this computational burden, the MML-EM algorithm can be adopted instead.

In the MML-EM algorithm, the general form of the first-order partial derivative with respective to ξ_*i*_ is given by

$$\frac{\partial \log L\left(\xi ;X\right)}{\partial \xi_{i}}=\sum_{s=1}^{S}\int \mathrm{Pr}\left(\theta |x_{s},\xi^{\left(\mathrm{old}\right)}\right)\frac{\partial \log\;\mathrm{Pr}{\left(Z_{i}=x_{si}|\theta ,\xi_{i}\right)}^{\chi \left(x_{si}\right)}}{\partial \xi_{i}}d\theta$$ (15)

[39, 54], which involves the posterior distribution conditioned on **x**_*s*_ and **ξ**^(old)^ and a score function of ξ_*i*_ (i.e., $\partial \log\;\mathrm{Pr}{\left(Z_{i}=x_{si}|\theta ,\xi_{i}\right)}^{\chi \left(x_{si}\right)}/\partial \xi_{i}$), where **ξ**^(old)^ is the estimates from the previous iteration. In practice, the following complete-data equations of gradient vector and Hessian matrix can be used to form a Newton-Raphson optimization scheme:

$$\frac{\partial \log L\left(\xi ;X\right)}{\partial \xi_{i}}=\sum_{q=1}^{Q}\sum_{z=0}^{C}r_{izq}\frac{\partial \log\left[\mathrm{Pr}\left(Z_{i}=z|V_{q},\xi_{i}\right)\right]}{\partial \xi_{i}},$$ (16)

$$\frac{\partial^{2}\log L\left(\xi ;X\right)}{\partial \xi_{i}^{2}}=\sum_{q=1}^{Q}\sum_{z=0}^{C}r_{izq}\frac{\partial^{2}\log\left[\mathrm{Pr}\left(Z_{i}=z|V_{q},\xi_{i}\right)\right]}{\partial \xi_{i}^{2}}\;(\mathrm{second}\;\mathrm{partial}\;\mathrm{derivative}),$$ (17)

$$\frac{\partial^{2}\log L\left(\xi ;X\right)}{\partial \xi_{i}\partial \xi_{i^{\prime }}}=\sum_{q=1}^{Q}\sum_{z=0}^{C}r_{izq}\frac{\partial^{2}\log\left[\mathrm{Pr}\left(Z_{i}=z|V_{q},\xi_{i},\xi_{i^{\prime }}\right)\right]}{\partial \xi_{i}\partial \xi_{i^{\prime }}}\;(\mathrm{cross}\;\mathrm{partial}\;\mathrm{derivative})$$ (18)

[39, 54, 55], where *Q* is the number of numerical quadrature points required for numerical integration, *V*_*q*_ is a quadrature point, *r*_*izq*_ is the expected frequency of response *z* for item *i* at *V*_*q*_ given by

$$r_{izq}=\sum_{s=1}^{S}\frac{\chi \left(x_{si}\right)n_{s}f\left(V_{q}\right)\prod_{i=1}^{I}\mathrm{Pr}\left(Z_{i}=x_{si}|V_{q},\xi_{i}^{\left(\mathrm{old}\right)}\right)}{\sum_{q=1}^{Q}f\left(V_{q}\right)\prod_{i=1}^{I}\mathrm{Pr}\left(Z_{i}=x_{si}|V_{q},\xi_{i}^{\left(\mathrm{old}\right)}\right)}.$$ (19)

For a wide variety of quasi-Newton optimization algorithms, providing only the gradient vector is adequate for estimation.

In the MML-EM algorithm, the posterior distribution of **θ** is computed given previous item estimates in the expectation step (E-step), followed by the maximization step (M-step) which is used to maximize the more manageable complete-data log-likelihood function with respect to item parameters given fixed *r*_*izq*_. The E-step and M-step are repeated successively until some termination criteria are satisfied (e.g., differences of estimates between iterations are smaller than 10^−4^). The MML-EM algorithm is widely used for the unidimensional models, and is the default estimation method in GGUM2004 and **mirt**.

## mirt description and how-to

**mirt** is a comprehensive psychometric package for multidimensional item response theory in **R**, which contains various model-based functions for fitting and analyzing IRT models. These features include: parameter estimation, item fit, person fit, model fit, reliability calculation, multilevel modeling, graphical output options, etc. [34]. Various MIRT models supported by **mirt** have been listed on the online manual of **mirt**. However, most of the internally optimized models are restricted to the family of dominance models. To inform researchers and practitioners that **mirt** not only supports dominance models, this section demonstrates that unfolding models can also be analyzed by controlling several of the more recent functional developments in the package. We aim to make these features in **mirt** more transparent to practitioners, and provide instructions regarding how to set up customized IRT models. We take the unfolding models, for instance, to illustrate the idea in the following, though strictly speaking the presentation is not limited solely to unfolding models.

To implement the estimation by **mirt** for non-native item probability functions, one must first build customized probability functions for the respective IRT models. First, the user must construct a single **R** function whose output is a probability matrix (where each row represents a given θ value and each column represents the respective response category) with three input arguments: a parameter vector, a matrix of quadrature points of θ, and the number of observed categories for the item. After this has been defined, a customized item type object can be created in the working environment with suitable starting values, parameter boundary constraints, analytical or numerical derivative computations for the MML-EM algorithm, and so on. A tutorial is given in the following empirical example, which can be replicated for other IRT models of the same form.

## An empirical example applying unfolding models

In this example, a classical unfolding dataset about attitudes towards capital punishment [41] is adopted for illustration purpose (see S1 Capital Punishment). The data for this example is publicly available at http://ggum.gatech.edu/cpdat.txt, and detailed descriptions of the items can be found at http://ggum.gatech.edu/capsdesc.html. In total there were 245 subjects in this dataset who indicated their attitudes towards capital punishment on multiple 6-point rating scale items, where 1 = Strongly Disagree, 2 = Disagree, 3 = Slightly Disagree, 4 = Slightly Agree, 5 = Agree and 6 = Strongly Agree. Previously, Roberts and Laughlin [41] conducted a preliminary analysis on these data by principal component analysis and found a two-factor solution with a simplex pattern of component loadings, which suggests the data is likely to respect the unidimensional unfolding mechanism [for more information, see 56]. Furthermore, Roberts and Laughlin [41] used the infit statistics [57] to heuristically screen poorly fit items, and subsequently retained only items 2, 9, 10, 12, 13, 14, 15, 16, 17, 18, 20, and 24 for subsequent analyses. After creating this subset of items, Roberts and Laughlin [41] selected the graded unfolding model (UM3) to fit to this data set.

In this example, we demonstrate how to replicate this analysis and compare the results from **mirt** and GGUM2004 based on the 12 retained items using the UM3 response model. The intention of this example analysis is to give readers back-reference for previously analyzed data, the appropriateness of using the open-source **mirt** package, and to provide a more structured description of how front-end users can define customized item response models in their own analyses.

### Writing a customized IRT model in mirt

First, the most general probability function—the GGUM (UM8)—for six-point items in **R** is defined and presented in the Step 1 of Supporting Information (see S1 R Syntax). As well, the GGUM can be reduced to the graded unfolding model (UM3) with appropriate constraints. The first observations to note is in regards to the three required input objects: the argument x is constructed to represent a vector for the respective parameters (e.g., x**[1]** is the δ, x**[2]** is the α, x**[3]** is the τ_1_, x**[4]** is the τ_2_, x**[5]** is the τ_3_, x**[6]** is the τ_4_, x**[7]** is the τ_5_), Theta is a matrix representing the values of **θ** and their quadrature instantiations (e.g., the rows reflect the quadrature and column the number of dimensions), and ncat is the number of categories.

For the MML-EM with Newton-based optimizers, one also has to provide the gradient vector and potentially the Hessian matrix of the probability function with respect to item parameters. The **mirt** package provides two approaches to accomplish this: one is to supply user-defined functions for calculating the analytical gradient and Hessian, and the other is to use numerical approximations (e.g., forward/central/Richardson extrapolation differentiation) or, if possible, symbolic evaluations. The former approach is primarily useful for speeding up computations of these required derivative functions, but also may be a step towards researchers formally contributing their customized models into the **mirt** package. The latter numerical or symbolic derivative approaches, on the other hand, can be used when no analytic gradient and Hessian have been defined because they are too cumbersome or error prone to derive explicitly. In this study, we adopt the quasi-Newton optimization algorithm in the M-step for its estimation stability, and because only the gradient functions are needed.

Continuing on, to create a customized unfolding model for **mirt**, one has to specify the name of model, initial values of parameters, parameter estimability logical values, and whether bounds are present in the parameter space. Step 2 illustrates the R code for these general definition steps. If the quasi-Newton method is preferred with symbolic derivatives, one has to either define the gradient function in **R,** or rely on the derivType = ‘symbolic’ argument to be passed when defining the model object; otherwise, the Richardson extrapolation approximation will be used by default, which while often slower will typically result it models as accurate and stable when symbolic methods are used. See Step 3 for R code. Finally, the defined R code, combined with the *createItem* function, can then be used to create the UM8 for **mirt**, as shown in Step 4.

For the starting values of unfolding models, it is often wise to assign appropriate signs of item locations, **δ**, based on the positive or negative descriptions of the items [4, 15, 24, 41, 58]. Although assigning signs of **δ** should be adequate, starting values of **δ** could be obtained by using correspondence analysis [56] from the **ade4** package [59]. Also, the sign of init_d also has to be modified based on the item contents. The syntax associated with obtaining and defining suitable starting values is shown in Step 5.

In situations where there are missing data present, the authors suggest temporarily using simple methods such as list-wise deletion or simple imputation when obtaining the associated starting values, because the magnitude of the starting values are less critical for estimation than the sign of the values. When ready, one can readily estimate the GGUM by passing the arguments to **mirt** to obtain the item and person estimates respectively shown in Step 6. In addition to UM8, one can readily estimate graded unfolding model (UM3). The details were shown in Step 7.

### Parameter estimates

**Fig 1:**
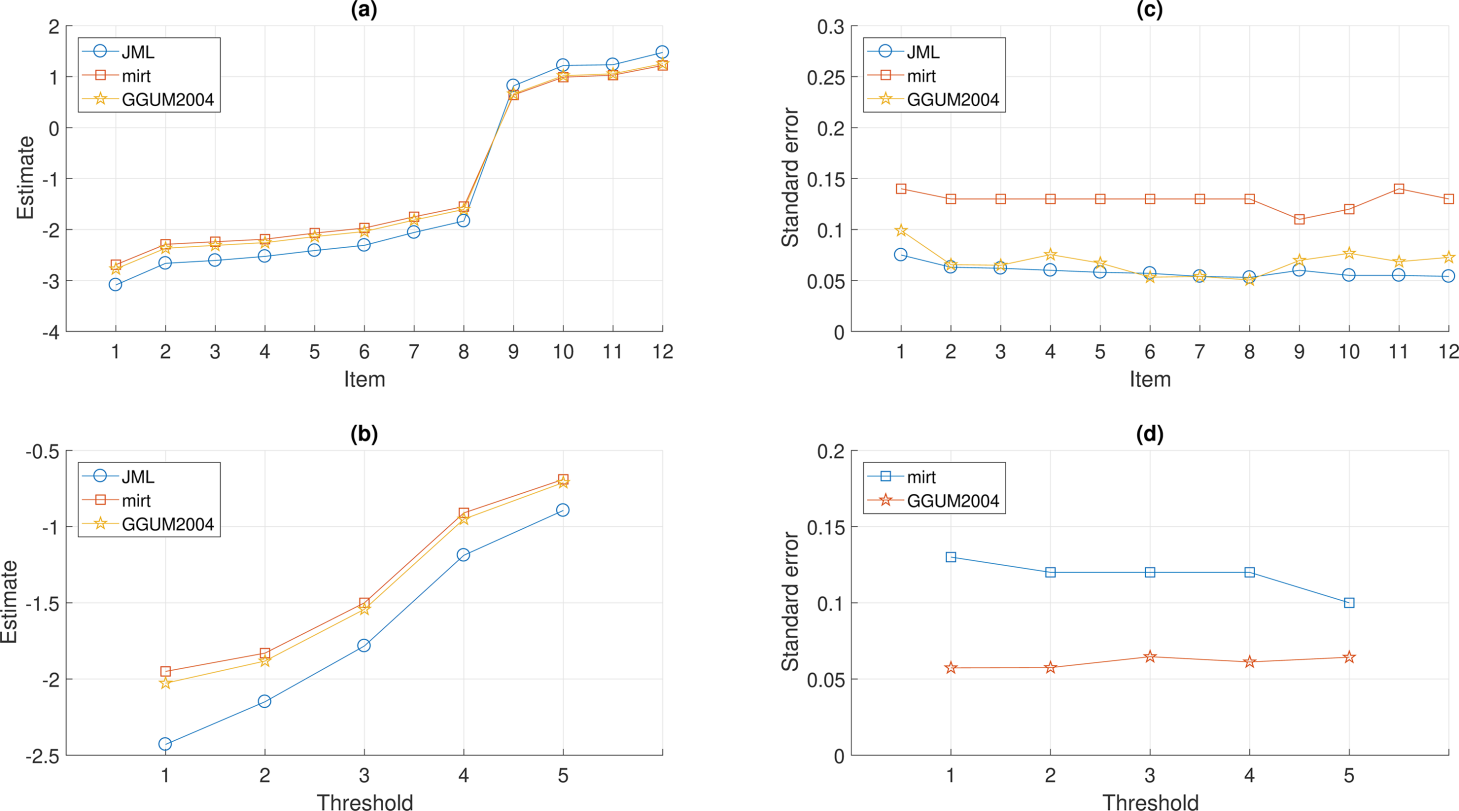
The estimates and standard errors of item locations and threshold parameters obtained from joint maximum likelihood estimation [JML; 41], mirt, and GGUM2004, for the capital punishment dataset of 245 respondents and 12 select items.

The results of item estimates and standard errors obtained from joint maximum likelihood estimation [JML; 41], **mirt**, and GGUM2004, were shown in Fig 1. Note that while the JML estimates were retrieved from Roberts and Laughlin (41), the mean of θ was rescaled to zero for comparison. Fig 1A and 1B indicate similar patterns of item estimates (δ and τ) among the three estimation criteria, where **mirt** and GGUM2004 yielded more similar results. Fig 1C and 1D show the JML estimation and GGUM2004, which used the empirical cross-product approach, tended to underestimate standard errors [60], whereas **mirt** yielded reliable standard error estimates by the more theoretically optimal Oakes identity approximation method [61]. The correlations of person estimates between **mirt** and GGUM2004 were .9998, implying that both software packages yielded nearly the same estimates. Note that Roberts and Laughlin [41] did not report standard error of thresholds for JML estimation.

### Further analyses by mirt

In addition to parameter estimates, **mirt** also provides several options for further analysis. For example, item-fit statistics can be computed by the *itemfit* function in **mirt** via, for example, *itemfit(mod)*, where *mod* is the model object in R obtained from *mirt* function (See Step 8). Empirical reliability coefficients can be readily obtained by *empirical_rxx(person)*, where *person* is an object in R that contains point estimates for **θ** and the associated standard error estimates via the *fscores* function. For UM3 in the above example, the reliability coefficient was found to be .89. The ICC of each item can also easily be obtained by calling *itemplot(mod*, *index)*, where *index* is the item index, or via *plot(mod*, *type)* to generate several plots for the test as a whole. Unfortunately, due to space constraints, we cannot exhaust all the options available in **mirt** within this study, but encourage readers to investigate the secondary analysis options currently supported by **mirt**. Specifically, interested readers should refer to the online manual of **mirt** (https://cran.r-project.org/web/packages/mirt/mirt.pdf) to discover many more options and features available.

## Numerical examples and simulations

Simulated data were used to investigate the parameter recovery via **mirt** under the **R** software environment. Here, the focus is to assess the recovery of item parameters where the latent trait is integrated (i.e., marginalized) out of the likelihood function. Once the estimates of item parameters are available, it is usual to estimate the individual’s estimates via maximum likelihood estimation, expected a posteriori, maximum a posteriori, and so on given the point-estimates of item parameters [55]. Therefore, the quality of individual estimates highly depend on how well the item parameters are recovered. With limited space, the following simulations cannot exhaust all possible conditions; however, the authors aim to demonstrate the utilities of **mirt** in regular empirical situations.

To begin, the performances of parameter recovery between **mirt** and GGUM2004 were compared based on the eight models found within GGUM2004 [25]. The purpose was to assess whether **mirt** could perform as well or better than the well-studied GGUM2004 software. Second, focus was on whether the parameter recovery of Luo’s [26] unidimensional unfolding models for Likert-scale data could also be obtained with sufficient accuracy. Lastly, Luo’s [40] multidimensional unfolding Likert-scale data with a between-item design was simulated so that the parameter recovery properties of **mirt** could be studied for these multidimensional models.

The overall assessment was determined by the bias and root-mean-square error (RMSE) of an estimator $\hat{\xi }$ computed by $R^{-1}\sum_{r=1}^{R}\left({\hat{\xi }}_{r}-\xi \right)$ and $\sqrt{R^{-1}\sum_{r=1}^{R}{\left({\hat{\xi }}_{r}-\xi \right)}^{2}}$, respectively, where ξ was the true parameter and *R* = 100 [20, 48, 49]. Other studies for the unfolding models have used as few as 30 replications [15, 20, 29, 41] or fewer [3, 62], however 100 replications appeared to be sufficient to obtain stable RMSE and bias estimates for comparison between the respective software packages.

In addition to parameter recovery, the behavior of the standard errors was studied for these respective models. The standard error of estimates can be obtained by numerically evaluating the observed data log-likelihood at a grid of points in the **ξ** space (e.g., forward, central, Richardson extrapolation, or the Oakes Identity Approximation method) in **mirt** [63]. Due to the heavy computation of the Monte Carlo studies, the authors used the central difference for unidimensional models and the forward difference for multidimensional models for illustration; however, front-end users should generally adopt the Oakes Identity method for its precision. The average of $SE({\hat{\xi }}_{r})$ across replications was compared with the empirical standard deviation of the estimator (i.e., $SD(\hat{\xi })=\sqrt{{RMSE}^{2}-{bias}^{2}}$), described by a relative measure (RM): $RM(\hat{\xi })=[R^{-1}\sum_{r=1}^{R}SE({\hat{\xi }}_{r})]{SD(\hat{\xi })}^{-1}-1$. Values of RE > 0 indicates the standard error is overestimated; otherwise, it is underestimated when RM < 0. RM close to 0 means the standard error is well estimated.

Complete syntax for all numeric examples are provided in S2 R Syntax. Finally, although the authors adopted the quasi-Newton method with analytical gradient vectors only (via symbolic differentiation) throughout simulation studies, an example of providing a user-defined analytical Hessian matrix function is also given in the tutorial for completeness.

### Example 1: Performances between mirt and GGUM2004

#### Design

The UM3 (graded unfolding model) and UM8 (GGUM) of GGUM2004 were adopted for simulating data, and were estimated by **mirt** and GGUM2004. The sample sizes studied were 250, 500, and 1,500, and the θ was generated from a standard normal distribution. The test length was 10 and 20, respectively. The true values of δ_*i*_ and τ_*ik*_ were generated consistent with the first simulation study of Wang, Liu [15]: the values of **δ** ranged from -2 to 2 with equal distance, and a four-point scale for every item were assumed for simplicity, where **τ** = (-1.10, -0.72, -0.30) for each item of UM3 and UM8. The true values of **α** were randomly generated within 0.76 and 1.34 for UM8 [15], whereas **α** = **1** for UM3. The MML-EM method was used, where the quadrature points were 50 ranging from -4 to 4 for GGUM2004 and **mirt**. In the maximization step, the GGUM2004 adopted the Newton-Raphson algorithm as the default, while the authors used a quasi-Newton method (via the *nlminb* solver) in **mirt**. The MML-EM was terminated early based on whether the absolute maximum difference of estimates between iterations fell below 0.0001 for GGUM2004 and **mirt** within 500 of possible EM iterations; otherwise, the data were discarded and resimulated.

#### Results

The maximum absolute values of biases and RMSEs for the parameter estimates are summarized in Table 1 when 10 items for UM3 and UM8. Overall, the maximum absolute values of biases and RMSEs for the parameter estimates were close to zero for UM3, except for UM8 when using 10 items estimated by GGUM2004. Other results for 20 items and 30 items were not shown here because the patterns of results were similar, but are available from the author upon request. Based on the observed behavior, it was evident that the bias and RMSEs were close between GGUM2004 and **mirt** for UM3; however, the performance of GGUM2004 was markedly worse than **mirt** for UM8. For instance, the maximum absolute value of bias and RMSE were respectively 0.327 and 0.790 for $\hat{\tau }$ of UM8 for GGUM2004 when sample size was 250, but they were only .056 and .480 for **mirt**. Note that the most severe bias and RMSE of the estimators were primarily associated with smaller sample size conditions.

**Table 1:** Maximum absolute values of the bias and root mean square error (RMSE) among items when 10 items were used in the Example 1.

	Bias			RMSE		
UM3 (graded unfolding model)						
Sample Size	250	500	1500	250	500	1500
$\hat{\delta }$							
**GGUM2004**	.018	.021	.015	.126	.089	.055
**mirt**	.018	.023	.013	.132	.096	.060
$\hat{\tau }$						
**GGUM2004**	.014	.019	.008	.082	.056	.031
**mirt**	.014	.021	.006	.096	.067	.035
${\hat{\sigma }}^{2}$						
**mirt**	.012	.021	.002	.136	.094	.053
UM8 (generalized graded unfolding model)						
$\hat{\alpha }$						
**GGUM2004**	.104	.039	.016	.292	.197	.114
**mirt**	.106	.043	.020	.288	.195	.115
$\hat{\delta }$						
**GGUM2004**	**.327**	**.207**	**.071**	**.722**	**.577**	**.280**
**mirt**	.056	.030	.022	.340	.252	.169
$\hat{\tau }$						
**GGUM2004**	**.323**	**.241**	**.076**	**.790**	**.630**	**.291**
**mirt**	.077	.059	.022	.480	.352	.204

**Fig 2:**
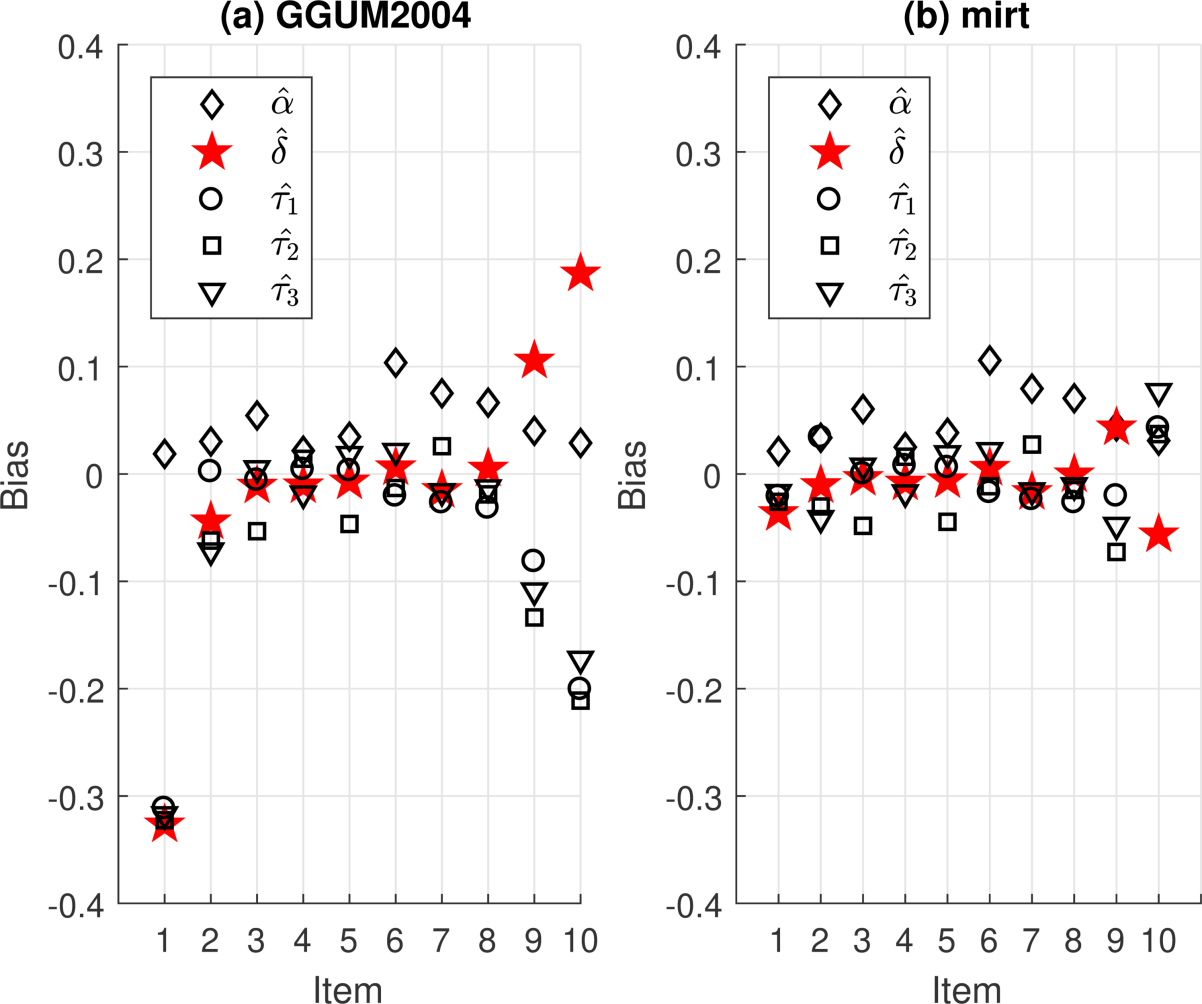
The bias values of parameter estimators of UM8 for 250 people and 10 four-point items from (a) GGUM2004 and (b) mirt.

It is interesting to compare all the bias estimates of the parameter estimators between GGUM2004 and **mirt** when there were only 250 observed responses and 10 items for UM8. To help illustrate these observations, the authors plotted the values of $\hat{\alpha },\;\hat{\delta }$ and $\hat{\tau }$ of UM8. Fig 2A shows the results estimated by GGUM2004. Trivially positive bias estimates were found for $\hat{\alpha }$, but the $\hat{\delta }$ and $\hat{\tau }$ were noticeably biased for both ends of items (i.e., relatively extreme items on the scale). The $\hat{\delta }$ estimations for extremely positive items tended to be biased more positively, where the $\hat{\delta }$ in the opposite tended to be biased more negatively. For $\hat{\tau }$, extreme items tended to be more negatively biased. These results may be explained due to sparse data in both extreme regions when coupled with the more unstable Newton-Raphson algorithm utilized in the M-step. This phenomenon has been rediscovered in the literature as well [25, 62]. The bias estimates were less severe for **mirt**, as shown in Fig 2B, which might be due to the stability of the select quasi-Newton method. When using larger sample size (e.g., 2000), the bias estimates were however reduced. Notably, to help with model stability, a practical approach has been suggested to regard ρ_*ik*_ = ρ_*k*_ equal across items (e.g., UM3) because the common scoring rubric is used for every item [15, 16, 41, 58, 62]. Overall, the results demonstrated that GUMM2004 provided more bias when estimating the eight studied unfolding models compared to **mirt** with the quasi-Newton method.

**Fig 3:**
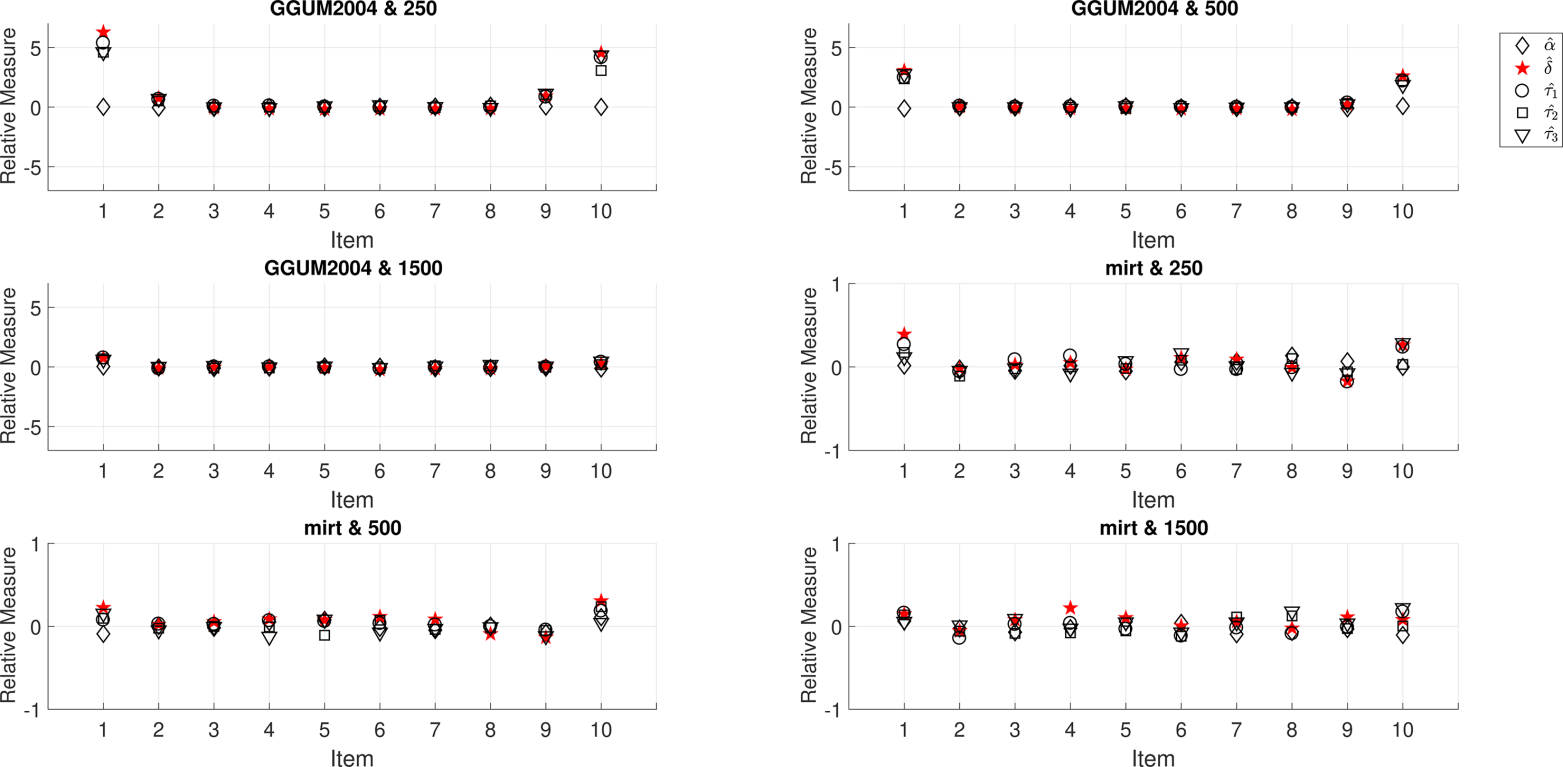
The relative measure of standard error estimators of UM8 for three sample sizes (250, 500, and 1,500) and 10 four-point items for GGUM2004 and mirt. Note. The y-axis limits are -7 and 7 for GGUM2004; the y-axis limits are -1 and 1 for mirt.

Regarding the performance of the standard error estimators, the results of UM8 were illustrated. For UM3, the results were omitted because the patterns were similar between the two programs. Fig 3 shows the results of RMs for three sample sizes (250, 500, and 1,500) given 10 items, respectively, for GGUM2004 and **mirt**. It is evident from these figures that GGUM2004 provided overestimated standard error estimates for both ends of items (e.g., RM = 6.28 for the $\hat{\delta }$ of the first item) when sample size was 250. Fortunately, the RM reduced as the sample size increased. In contrast, the overall RM for **mirt** ranged from -0.18 to 0.39 for all parameters, generally indicating that the standard errors were properly reflecting the sampling variability of the parameter estimates.

For latent trait recovery, the correlation of true values and estimates (MLEs or EAPs) for **mirt** was around .96 under all conditions, and the correlation was also around .96 (only for EAP) for GGUM2004. This implies that both programs can effectively recover the linear relationship between true values and estimates. Overall, based on the performances of item parameter and standard error estimation, **mirt** appears to be a suitable alternative to GGUM2004 for parameter estimation of the unfolding models studied.

### Example 2: Unidimensional unfolding models with different operational function designs

In this numerical example, the parameter recoveries of unidimensional unfolding models were investigated. The five models in question were derived from the HCM and the GUM. For consistency, the simulation setting was almost identical to Example 1: sample size (250, 500, and 1,500), standard normal distribution for latent trait, test length (10 and 20), values of δ ranging from -2 to 2 with equal distance, four-point scales, and the values of α were randomly sampled within 0.76 and 1.34 (19). The **ρ** = (1.102, 0.794, 0.587) was adopted which was used in the work of Wang et al. [19]. With the common scoring rubric, only a set of common thresholds across items were selected to be estimated—that is, ${\hat{\rho }}_{ik}={\hat{\rho }}_{k}$ [15, 41, 62]. The arguments of **mirt** were the same as in Example 1.

#### Results

Table 2 represents the maximum absolute values of the bias and RMSE estimates for the item parameters. The absolute maximum values of bias were smaller than 0.128 in all conditions, whereas the absolute maximum values of RMSEs were all smaller than 0.446. Higher sample sizes tended to lower the RMSEs (i.e., lower the sampling error). Also, longer test lengths resulted in slightly lower RMSEs. For the condition of 10 items and sample size 1,500, the maximum absolute values of the bias and RMSE for the GUM were 0.011 and 0.124 for $\hat{\delta }$, 0.018 and 0.068 for $\hat{\alpha }$, and 0.014 and 0.044 for $\hat{\rho }$, which could be compared with the results of Table 1 (under Condition 1) of Wang, Liu [15], who obtained the maximum absolute values of bias and RMSE of 0.033 and 0.066 for $\hat{\delta }$, 0.050 and 0.089 for $\hat{\alpha }$, and 0.022 and 0.038 for $\hat{\rho }$, from their Bayesian MCMC estimation approach. Although the two results are not based on the same replicated data or estimators, but rather the same ‘true’ item parameters, the comparison indicates that **mirt** performs very similar to the MCMC estimation. For other models, the results are similar to those of the GUM. Overall, the parameter recovery appeared to be satisfactory.

**Table 2:** Maximum absolute values of the bias and root mean square error (RMSE) among items for five models in the Example 2.

	Bias						RMSE					
Test length	10			20			10			20		
Sample size	250	500	1500	250	500	1500	250	500	1500	250	500	1500
$\hat{\delta }$												
HCM	.128	.017	.019	.078	.030	.035	.446	.298	.172	.326	.228	.135
GUM	.071	.010	.011	.043	.021	.025	.305	.202	.124	.263	.168	.095
$\hat{\alpha }$												
HCM	.053	.035	.018	.069	.042	.025	.212	.151	.088	.213	.149	.080
GUM	.053	.032	.018	.058	.035	.020	.160	.109	.068	.186	.128	.069
$\hat{\rho }$												
HCM	.050	.029	.012	.041	.020	.009	.139	.101	.063	.112	.071	.040
GUM	.056	.028	.014	.033	.023	.009	.117	.079	.044	.075	.058	.029

The RM for the standard error estimator ranged from -0.19 to 0.20 for the five models under the three sample sizes with 10 items, and ranged from -0.31 to 0.35 with 20 items. Regarding the trait recovery, the correlation of true values and estimates (MLEs) for **mirt** ranged from 0.92 to 0.99 for the five models under the three sample sizes with 10 items, and it ranged from 0.96 to 0.99 with 20 items. These ranges were similar to the results in Example 1. Thus, the trait correlation estimator and the standard error estimator of **mirt** were acceptable for the five models.

### Example 3: Multidimensional unfolding models

#### Design

The numerical example here investigates the parameter recovery of a class of multidimensional unfolding models for Likert-scale items. Two multidimensional unfolding models were considered for the simulation: MHCM, and MGUM, which are multidimensional versions of HCM and GUM. Because the parameter estimation of these models have not been investigated in the literature, the simulation settings were set similar to the work of Wang and Wu [20]. A three-dimension design with between-item responses [47] was adopted for illustrative purpose; that is, each item only measured a single latent trait. Two test lengths were used, 7 and 14, for each dimension [20]. Regarding item parameters, δ ranged between -2 and 2 with equal steps; **ρ** = (1.102, 0.794, 0.587) for each dimension; the value of α was sampled randomly from the range from 0.76 to 1.34, which are the same settings as Example 2. There were 500 and 1,500 randomly drawn latent traits which were sampled from a multivariate normal distribution with **μ** = **0** and

$$\Sigma =\left[\begin{array}{ccc} 1 & \rho_{21} & \rho_{31}\\ \rho_{21} & 1 & \rho_{32}\\ \rho_{31} & \rho_{32} & 1 \end{array}\right],$$ (20)

which represents the true correlations between dimensions. For simplicity, all correlations were organized to be equal, and were set to either 0, .4, and .8, respectively in three respective conditions [58]. For estimation purposes, however, the three correlations were freely estimated. Although zero correlation rarely occurs in practice, it was included to serve as a performance baseline. The means and variances of **θ** were set to zero and one, respectively, for model identification. Finally, although selecting the number of quadrature point is an empirical question, and should be increased if the accuracy incurred by numerical integration is too low, the default number of quadrature points was set equal to 15 per dimension (i.e., 3,375 in total) by **mirt**.

#### Results

The maximum absolute values of the bias and RMSE estimates are shown in Table 3. Comparing the absolute maximum values of bias and RMSE between sample size 500 and 1,500 for the five models, the estimates were overall slightly lower for the larger sample size. For sample size 500 and test length 7 for the three correlation conditions, the highest bias value was .068 for the $\hat{\alpha }$ of MGUM, whereas for sample size 500 and test length 14 the highest bias value was .050 for $\hat{\delta }$ of MHCM. In terms of RMSEs, the $\hat{\delta }$ of MHCM tended to have higher sampling variability. The highest value was .380 when sample size 500 and test length 7 when correlation was zero. For sample size 1,500 and test length 7 for the three correlation conditions, the highest RMSE value was .184 for the $\hat{\delta }$ of MHCM, whereas for sample size 1,500 and test length 14 the highest RMSE estimate was .173 for $\hat{\delta }$ of MHCM. The bias range of correlation estimates for all conditions (not shown in Table 3) was between 0.000 and 0.041, whereas the RMSE range was between 0.012 and 0.073. Overall, the performance of **mirt** with respect to recovering the parameters for the five models appeared to be satisfactory.

**Table 3:** Maximum absolute value of the bias values and root mean square error (RMSE) among items for five multidimensional unfolding models in the Example 3.

	Bias											
Sample size	500						1,500					
Test length	7			14			7			14		
Correlation	0	.4	.8	0	.4	.8	0	.4	.8	0	.4	.8
$\hat{\delta }$												
MHCM	.056	.060	.045	.046	.045	.050	.027	.034	.028	.037	.030	.037
MGUM	.033	.056	.044	.040	.033	.041	.028	.036	.045	.026	.022	.023
$\hat{\alpha }$												
MHCM	.064	.067	.050	.039	.040	.029	.027	.031	.025	.041	.033	.023
MGUM	.052	.068	.054	.034	.037	.027	.030	.031	.027	.033	.027	.022
$\hat{\rho }$												
MHCM	.047	.033	.037	.027	.019	.018	.014	.011	.014	.012	.008	.007
MGUM	.036	.022	.028	.019	.019	.021	.010	.011	.012	.008	.006	.005
	RMSE											
$\hat{\delta }$												
MHCM	.380	.327	.275	.293	.285	.277	.184	.170	.153	.173	.162	.147
MGUM	.225	.216	.195	.183	.187	.187	.120	.123	.114	.109	.109	.104
$\hat{\alpha }$												
MHCM	.225	.212	.205	.167	.170	.152	.107	.112	.113	.106	.093	.081
MGUM	.181	.171	.157	.136	.141	.122	.105	.097	.087	.083	.078	.069
$\hat{\rho }$												
MHCM	.137	.130	.157	.085	.088	.084	.075	.077	.070	.053	.055	.050
MGUM	.100	.104	.097	.054	.057	.055	.052	.056	.051	.033	.032	.036

The RM for the standard error estimator ranged from -0.29 to 0.50 for the five models under all the conditions. Regarding the trait recovery, the correlation of true values and estimates (MLEs) for **mirt** ranged from 0.80 to 0.97 for the two models under all the conditions. For the EAPs, the correlation estimates ranged from 0.87 to 0.97, which were slightly larger than the MLEs because a correct Gaussian prior distribution was used for the EAPs.

## Concluding remarks

Unfolding models are suitable when the underlying measurement process contains a proximity property with respect to the item-level stimuli. Although they have attracted huge attention recently [9, 12, 15, 20, 64], the development of parameter estimation software for various unfolding models has largely been left behind. To enhance the utilities of unfolding models in practice, the **mirt** package was adopted in this article and evaluated using Monte Carlo simulation studies. Overall results show that the parameters can be well recovered in a number of known simulation conditions for several unfolding models. The numerical examples and simulations explored also provided partial evidence that the **mirt** package can serve as an alternative to the GGUM2004 software. As was also apparent, Luo’s unidimensional and multidimensional models can be well estimated by **mirt** for Likert-scale data. Although the remaining six models in GGUM2004, as well as the ALM, SSLM, and PARELLA, were not presented in the above simulation studies, our preliminary study suggests that the parameters in these respective models can also be recovered well. These results are available from the authors upon request.

Regarding future applications with the parameters of unfolding models estimated using **mirt**, researchers could also feasibly begin to construct an item bank for administering online real-time scoring [65]. Relatedly, the application of the MUM to computerized adaptive testing and computerized classification testing is of great value in practice. With the advent of computers and apps on smartphones, tablets, and other portable devices, the survey time is often greatly reduced, and therefore multidimensional tests can be constructed with maximum precision [66]. Relevant developments of item selection algorithms and classification strategies are still open area for the MUMs.

With space limitations, the authors only provide a profile of **mirt** for parameter estimation of unfolding models. However, there are a number of post-hoc analysis functions available in **mirt** package that analysts will often also be interested in, which are also supported whenever customized IRT models have been defined. For instance, item-fit statistics such as Zh values [67], S-X2 statistics [68], Stone’s X2* [69] and the PV-Q1 statistics [70], model-fit with M2 [71], person estimates, plotting methods, and so on are available for assessing the quality of items and overall model in the analysis. Interested readers in these topics should refer to the online manual of **mirt** package. As it stands, however, the S-X2 statistics may require some modifications for unfolding models [72].

Another interesting area of future research involves studying and modeling pairwise preference response data. An IRT unfolding model proposed by Andrich [3] is specifically appropriate for this type of comparative data. Though naturally applicable to these types of data, the unfolding pairwise preference models are seldom used in the literature as well, which again may be due to the absence of available software. Thus, using **mirt** to estimate the parameters of pairwise unfolding models is left for further study, but is another area where **mirt** may be of substantial practical use.

In this article, the authors echo Luo’s [42] need for additional general computer programs that are useful for unfolding analyses. The authors demonstrated the utilities of **mirt** to estimate Likert-scale data following various unfolding models. Based on the simulations studied and example code provided, we recommend that researchers and practitioners adopt the **mirt** package in their own item response modeling work whenever they are interested in investigating both common and less common unfolding models. Although the features demonstrated in this article are new to the **mirt** software package, the current estimation functions for constructing and analyzing customized item response models clearly provide users with a powerful level of flexibility which ought to be adopted by practitioners and further studied in subsequent bodies of simulation-based research.

## Supporting information

S1 Fig The probability of endorsement for binary responses. (TIF)

S2 Fig The corresponding Fisher information function of θ, where δ = 0 and ρ = 1 for ALM, SSLM, PARELLA, HCM, and GUM. (TIF)

S3 Fig The two-dimensional hyperbolic cosine model’s probability function of θ_1_ and θ_2_ for a four-point Likert-scale item of α = (1,1), δ = (0, 0), and ρ = (3, 2, 1), where the three bold circles represent the threshold locations. Arrows annotate the regions of four categories.
(TIF)

S4 Fig The Fisher information function of θ_1_ from two views. The Fisher function of θ_2_ is similar to that of θ_1_, and omitted here.
(TIF)

S5 Fig The Fisher information function of θ_1_ from a single view. The Fisher function of θ_2_ is similar to that of θ_1_, and omitted here.
(TIF)

S1 Capital Punishment The classical unfolding dataset about attitudes towards capital punishment. (DAT)

S1 R Syntax Syntax of mirt for estimating parameters of generalized graded unfolding model (GGUM; Roberts, Donoghue, & Laughlin, 2000) for capital punishment dataset of six-point Likert scale. (DOCX)

S2 R Syntax Syntax of mirt for estimating parameters of generalized graded unfolding model (GGUM; Roberts, Donoghue, & Laughlin, 2000) for four-point Likert scale. Syntax of mirt for estimating parameters of unidimensional graded unfolding model (GUM; Luo, 2001) for four-point Likert scale. Syntax of mirt for estimating parameters of three-dimensional graded unfolding model for four-point Likert scale.
(DOCX)
