# Supplementary figures and images for: Fitting item response unfolding models to Likert-scale data using mirt in R
Source: PLoS One. 2018 May 3;13(5):e0196292. doi: 10.1371/journal.pone.0196292 (PMC5933773; doi:10.1371/journal.pone.0196292)

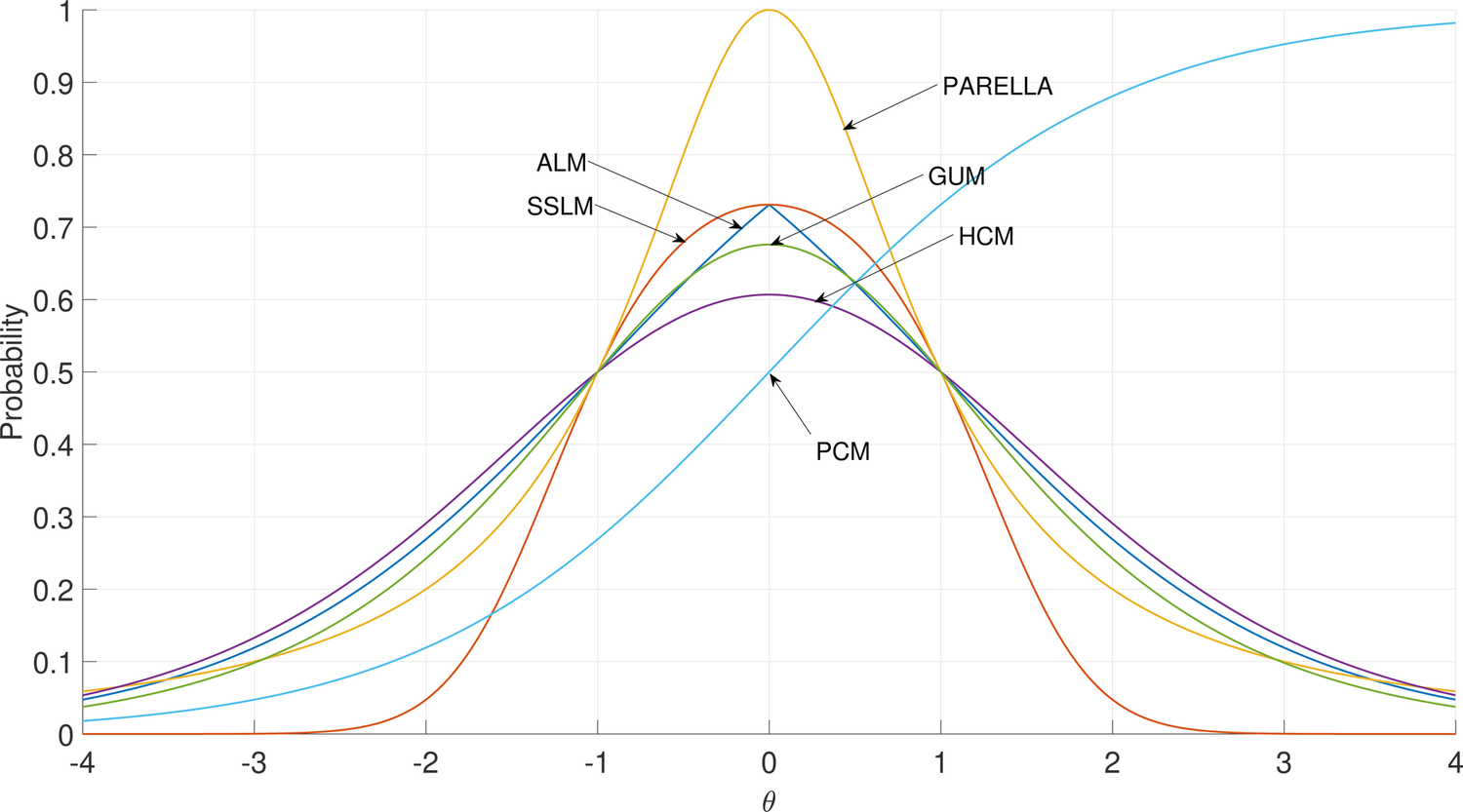

Supplement: S1 Fig — (TIF) [file pone.0196292.s001.tif]

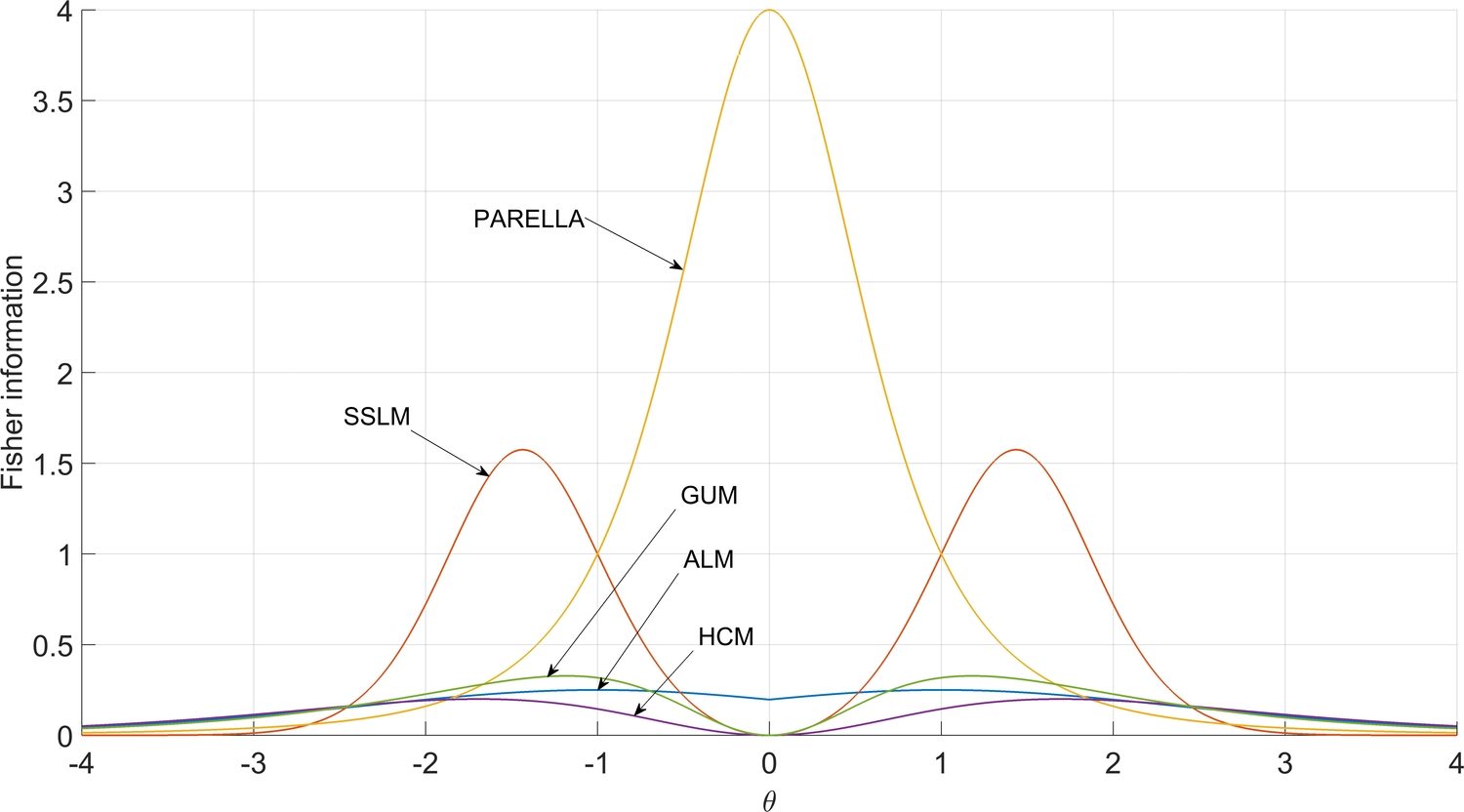

Supplement: S2 Fig — (TIF) [file pone.0196292.s002.tif]

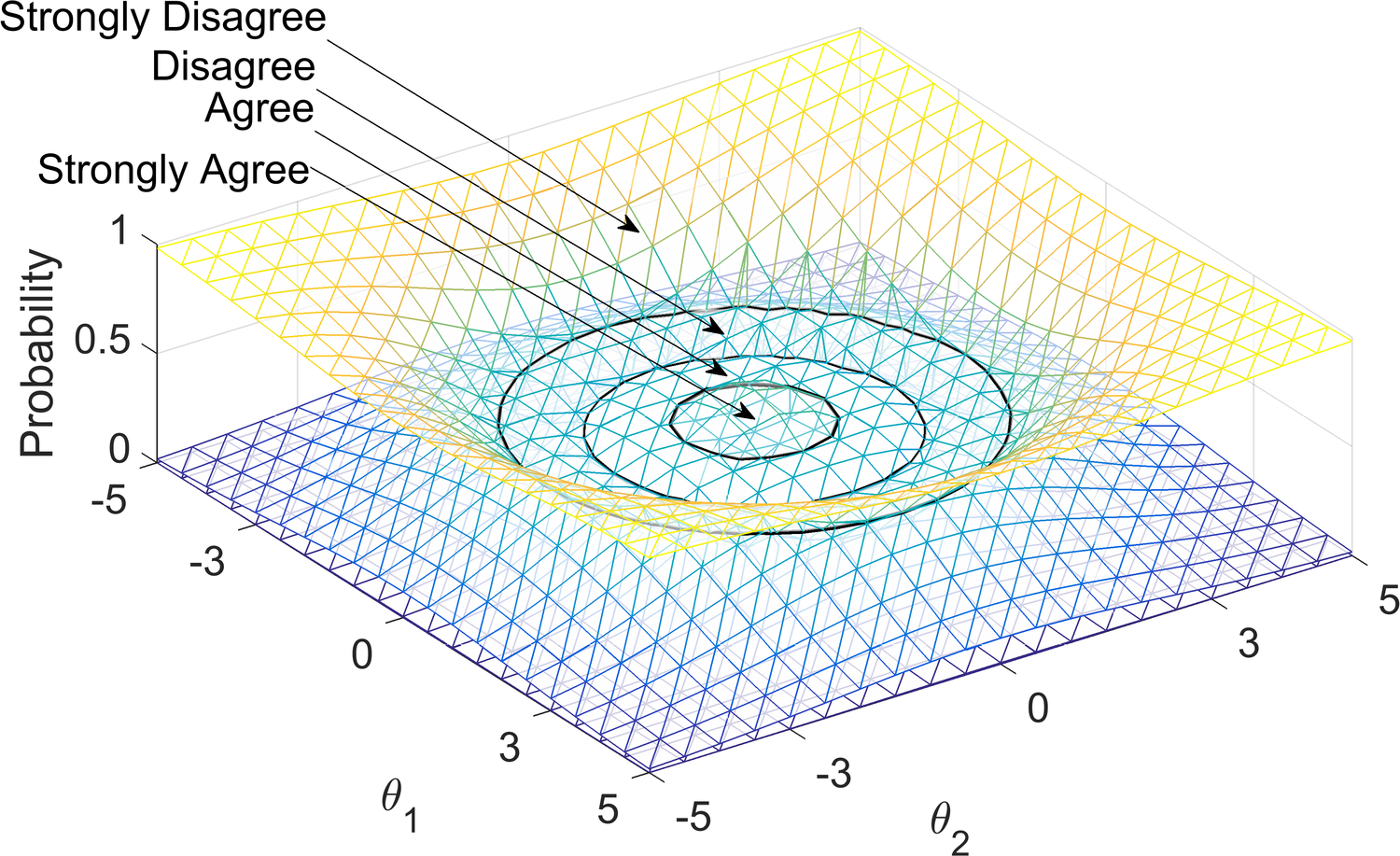

Supplement: S3 Fig — Arrows annotate the regions of four categories. (TIF) [file pone.0196292.s003.tif]

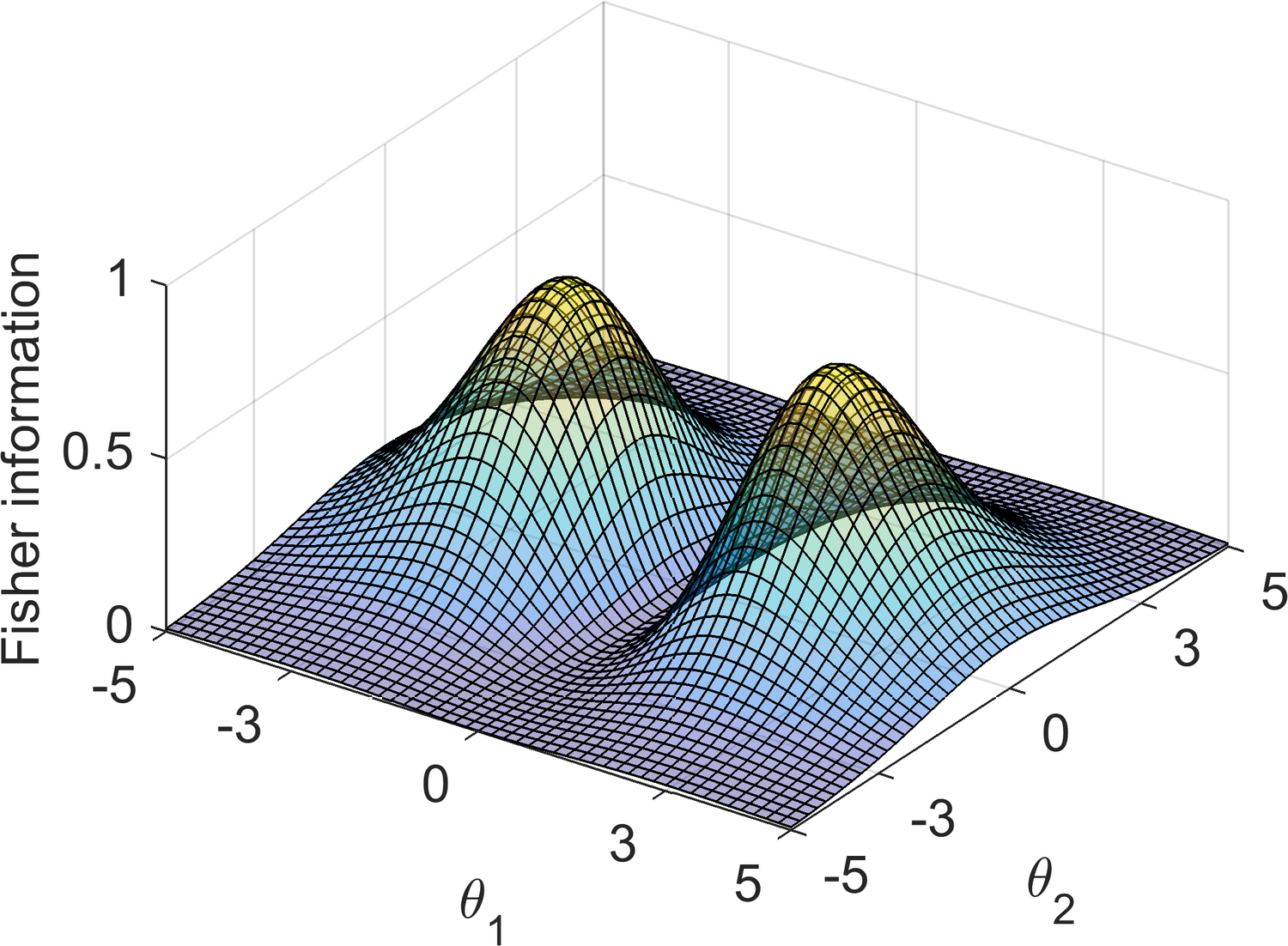

Supplement: S4 Fig — The Fisher function of θ2 is similar to that of θ1, and omitted here. (TIF) [file pone.0196292.s004.tif]

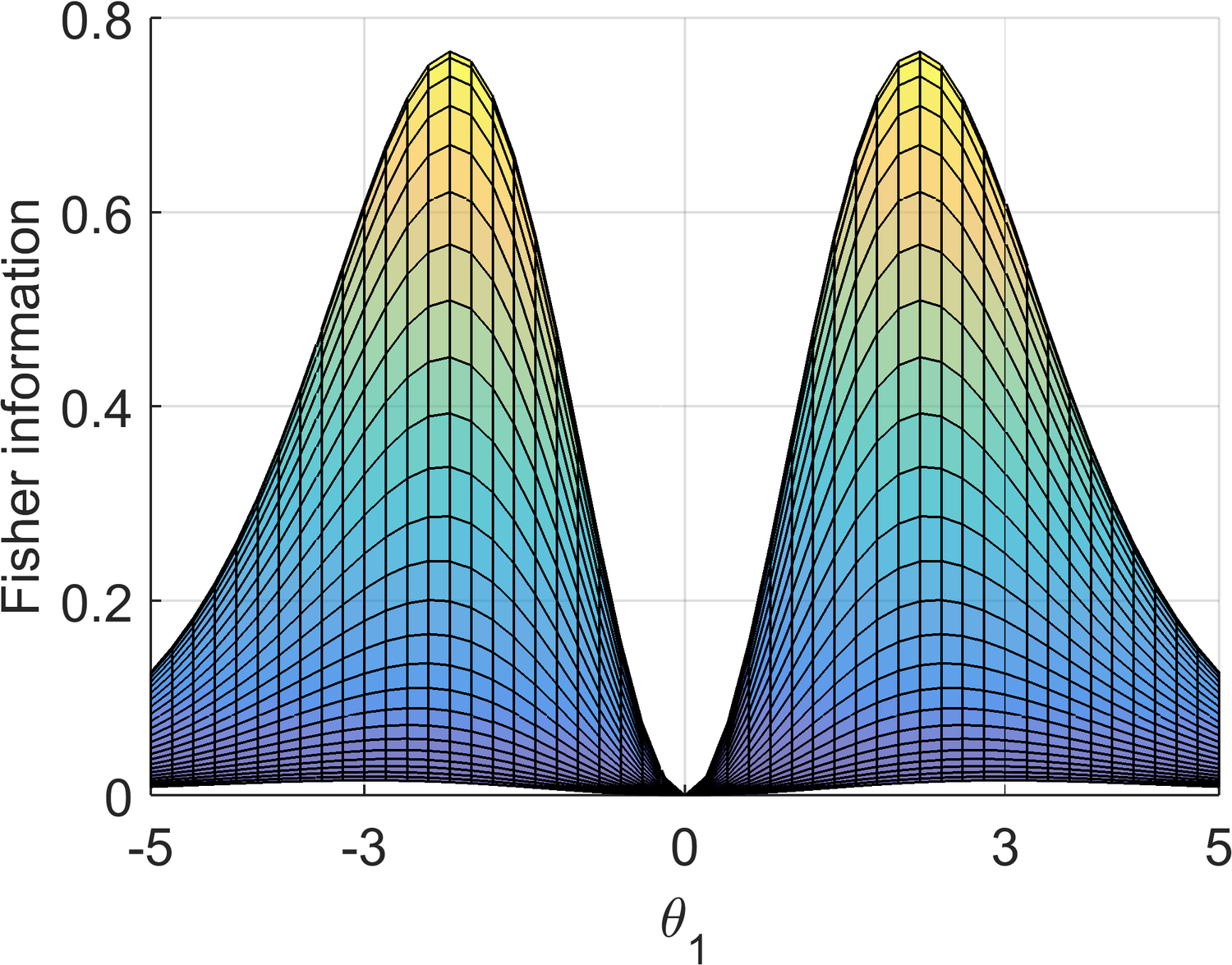

Supplement: S5 Fig — The Fisher function of θ2 is similar to that of θ1, and omitted here. (TIF) [file pone.0196292.s005.tif]
