# Supplementary material for: Fitting item response unfolding models to Likert-scale data using mirt in R
Source: PLoS One. 2018 May 3;13(5):e0196292. doi: 10.1371/journal.pone.0196292 (PMC5933773; doi:10.1371/journal.pone.0196292)
Supplement: S1 R Syntax — (DOCX) [file pone.0196292.s007.docx]

**R** syntax of **mirt** for estimating parameters of generalized graded unfolding model and/or graded unfolding model for Capital Punishment dataset of six-point Likert scale

**Step 0: Read data**

**R>** res <- read.table(file="YourData.txt")

**R>** dimnames(res) <- list(rownames(res, do.NULL = FALSE, prefix = "row"),colnames(res, do.NULL = FALSE, prefix = "Item."))

**R>** itemnum <- ncol(res) # number of items

**R>** C <- 6-1 # number of category minus one

The user has to replace “YourData.txt” with the file name of the data matrix whose size is sample size × number of items.

**Step 1: Write up the probability function**

**R>** P **<-** **function** **(**x, Theta, ncat**)** **{**

nu1 **<-** 1 **+** exp**(**x**[**2**]** ***** **(**2 ***** C **+** 1 **-** 0**)** ***** **(**Theta **-** x**[**1**]))**

nu2 **<-** exp**(**x**[**2**]** ***** **((**Theta **-** x**[**1**])** **-** x**[**3**]))** **+** exp**(**x**[**2**]** ***** **((**2 ***** C **+** 1 **-** 1**)** ***** **(**Theta **-** x**[**1**])** **-** x**[**3**]))**

nu3 **<-** exp**(**x**[**2**]** ***** **(**2 ***** **(**Theta **-** x**[**1**])** **-** x**[**3**]** **-** x**[**4**]))** **+** exp**(**x**[**2**]** ***** **((**2 ***** C **+** 1 **-** 2**)** ***** **(**Theta **-** x**[**1**])** **-** x**[**3**]** **-** x**[**4**]))**

nu4 **<-** exp**(**x**[**2**]** ***** **(**3 ***** **(**Theta **-** x**[**1**])** **-** x**[**3**]** **-** x**[**4**]** **-** x**[**5**]))** **+** exp**(**x**[**2**]** ***** **((**2 ***** C **+** 1 **-** 3**)** ***** **(**Theta **-** x**[**1**])** **-** x**[**3**]** **-** x**[**4**]** **-** x**[**5**]))**

nu5 **<-** exp**(**x**[**2**]** ***** **(**4 ***** **(**Theta **-** x**[**1**])** **-** x**[**3**]** **-** x**[**4**]** **-** x**[**5**]** **-** x**[**6**]))** **+** exp**(**x**[**2**]** ***** **((**2 ***** C **+** 1 **-** 4**)** ***** **(**Theta **-** x**[**1**])** **-** x**[**3**]** **-** x**[**4**]** **-** x**[**5**]** **-** x**[**6**]))**

nu6 **<-** exp**(**x**[**2**]** ***** **(**5 ***** **(**Theta **-** x**[**1**])** **-** x**[**3**]** **-** x**[**4**]** **-** x**[**5**]** **-** x**[**6**]** **-** x**[**7**]))** **+** exp**(**x**[**2**]** ***** **((**2 ***** C **+** 1 **-** 5**)** ***** **(**Theta **-** x**[**1**])** **-** x**[**3**]** **-** x**[**4**]** **-** x**[**5**]** **-** x**[**6**]** **-** x**[**7**]))**

de **<-** nu1 **+** nu2 **+** nu3 **+** nu4 **+** nu5 **+** nu6

cbind**(**nu1**/**de, nu2**/**de, nu3**/**de, nu4**/**de, nu5**/**de, nu6**/**de**)**

**}**

**Step 2: Set up mirt**

**R>** require**(**"mirt"**)**

**R>** name = "GGUM"

**R>** para = c(delta=0,alpha=2,rho1=-1.2,rho2=-1,rho3=-0.8,rho4=-0.7,rho5=-0.6)

**R>** toEst = rep(TRUE, 7)

**R>** lbound = c(-Inf,1e-10,rep(-Inf,5)) # lower bound

**Step 3: (Optional) Write up gradient function**

This step is optional in case users want to write their own analytical gradient function. Otherwise, numerical or symbolic approaches are supported.

For example, in the initial run use

**R>** require**(**"Deriv"**)**

**R>** dp1 **<-** Deriv**(**P,c**(**x**=**1,x**=**2,x**=**3,x**=**4,x**=**5,x=6,x=7**))**

to obtain the functions of the first-order derivatives symbolically. In practice, it is time-saving to write the P, and dp1, to a file for subsequent use:

**R>** fun **<-** list**(**f**=**P,dp1**=**dp1**)**

**R>** save**(**fun,file**=**"UM8"**)**

After these are saved to the hard-drive, load**(**"UM8"**)** will reload the functions for later use. Below these symbolic evaluations are included in the gradient function definition for createItem.

**R>** gr **<-** **function(**x, Theta**){**

P **<-** fun**$**f**(**x@par, Theta**)**

xLength **<-** length**(**x@par**)**

ThetaLength **<-** length**(**Theta**)**

r_P **<-** x@dat **/** P

dp1 **<-** array**(**fun**$**dp1**(**x@par, Theta**)**, c**(**ThetaLength,x@ncat,xLength**))**

grad **<-** numeric**(**length**(**x@par**))**

**for** **(**i **in** 1**:**xLength**){**

grad**[**i**]** **<-** sum**(**r_P ***** dp1**[**,,i**])**

**}**

grad

**}**

**Step 4: Create model**

**R>** ci <- createItem(name,par=para,est=toEst,P=fun$f, lbound=lbound,gr=gr)

If the gr input is omitted then the function will use Richardson extrapolation to compute the respective derivative terms by default. To obtain the derivatives symbolically, however, users may pass the argument derivType = ‘symbolic’ to override the default ‘Richardson’ method.

**Step 5: Check/set model’s starting values**

**R>** sv <- mirt(res,1,c(rep(name,itemnum)),customItems=list(GGUM=ci), pars='values')

**R>** library("ade4")

**R>** fit2 <- dudi.coa(res, scannf = FALSE, nf = 2)

**R>** init_d <- scale(fit2$c1[,1])[,1]

**R>** sv$value[sv$parnum[sv$name=='delta']] <- init_d

**Step 6: Run model**

**R>** mod <- mirt(res,1,technical=list(theta_lim=c(-4,4))

,quadpts=50,pars=sv,c(rep(name,itemnum)),customItems=list(GGUM=ci) ,SE**=TRUE**,SE.type**=**"Oakes",optimizer="nlminb",control=list(rel.tol=1e-4,iter.max=10),TOL=1e-4)

**R>** coef(mod, simplify = TRUE)

**R>** fscores(mod, method = "EAP",full.scores.SE=TRUE)

**Step 7: (Optional) Run submodels**

For UM7, add the following syntax to record the index in the structure table (i.e., ‘sv’):

**R>** indrho1 <- sv$parnum[sv$name == 'rho1']

**R>** indrho2 <- sv$parnum[sv$name == 'rho2']

**R>** indrho3 <- sv$parnum[sv$name == 'rho3']

**R>** indrho4 <- sv$parnum[sv$name == 'rho4']

**R>** indrho5 <- sv$parnum[sv$name == 'rho5']

and add the following argument in **mirt**:

constrain = list(c(indrho1),c(indrho2),c(indrho3),c(indrho4),c(indrho5)).

Note that constraints can be defined using the mirt.model() syntax format as well.

As a result of these constraint definitions, **mirt** will constrain the thresholds to be the same across items. For UM4, one just has to constrain the α*_i_* = 1 for all items and then allow for the variance of θ to be estimated:

**R>** sv$est[sv$name=="alpha"] <- FALSE

**R>** sv$value[sv$name=="alpha"] <- 1

**R>** sv[sv[,"name"]=="COV_11","est"] <- TRUE

For UM3, the above constraints of UM7 and UM4 have to be used simultaneously. For UM6 and its sub-models (UM1, UM2, UM5), they can be done in the same manner as above.
