# Supplementary material for: Fitting item response unfolding models to Likert-scale data using mirt in R
Source: PLoS One. 2018 May 3;13(5):e0196292. doi: 10.1371/journal.pone.0196292 (PMC5933773; doi:10.1371/journal.pone.0196292)
Supplement: S2 R Syntax — Syntax of mirt for estimating parameters of unidimensional graded unfolding model (GUM; Luo, 2001) for four-point Likert scale. Syntax of mirt for estimating parameters of three-dimensional graded unfolding model for four-point Likert scale. (DOCX) [file pone.0196292.s008.docx]

**R** syntax of **mirt** for estimating parameters of generalized graded unfolding model (GGUM; Roberts, Donoghue, & Laughlin, 2000) for four-point Likert scale

res**<-**read.table**(**file**=**"res.txt"**)**

dimnames**(**res**)** **<-** list**(**rownames**(**res, do.NULL **=** **FALSE**, prefix **=** "row"**)**,colnames**(**res, do.NULL **=** **FALSE**, prefix **=** "Item."**))**

itemnum **<-** ncol**(**res**)**

C **<-** 4**-**1

fun **<-** **function** **(**x, Theta, ncat**)** **{**

nu1 **<-** 1 **+** exp**(**x**[**2**]** ***** **(**2 ***** C **+** 1 **-** 0**)** ***** **(**Theta **-** x**[**1**]))**

nu2 **<-** exp**(**x**[**2**]** ***** **((**Theta **-** x**[**1**])** **-** x**[**3**]))** **+** exp**(**x**[**2**]** ***** **((**2 ***** C **+** 1 **-** 1**)** ***** **(**Theta **-** x**[**1**])** **-** x**[**3**]))**

nu3 **<-** exp**(**x**[**2**]** ***** **(**2 ***** **(**Theta **-** x**[**1**])** **-** x**[**3**]** **-** x**[**4**]))** **+** exp**(**x**[**2**]** ***** **((**2 ***** C **+** 1 **-** 2**)** ***** **(**Theta **-** x**[**1**])** **-** x**[**3**]** **-** x**[**4**]))**

nu4 **<-** exp**(**x**[**2**]** ***** **(**3 ***** **(**Theta **-** x**[**1**])** **-** x**[**3**]** **-** x**[**4**]** **-** x**[**5**]))** **+** exp**(**x**[**2**]** ***** **((**2 ***** C **+** 1 **-** 3**)** ***** **(**Theta **-** x**[**1**])** **-** x**[**3**]** **-** x**[**4**]** **-** x**[**5**]))**

de **<-** nu1 **+** nu2 **+** nu3 **+** nu4

cbind**(**nu1**/**de, nu2**/**de, nu3**/**de, nu4**/**de**)**

**}**

require**(**"mirt"**)**

name **=** "GGUM"

para **=** c**(**delta**=**0,alpha**=**1,rho1**=-**1,rho2**=-**1,rho3**=-**1**)**

toEst **=** rep**(TRUE**, 5**)**

lbound **=** c**(-Inf**,1e**-**10,rep**(-Inf**,3**))** # lower bound

ci **<-** createItem**(**name,par**=**para,est**=**toEst,P**=**fun**$**f, lbound**=**lbound, derivType=’symbolic’**)**

sv **<-**mirt**(**res,1,c**(**rep**(**name,itemnum**))**,customItems**=**list**(**GGUM**=**ci**)**, pars**=**'values'**)**

library**(**"ade4"**)**

fit2 **<-** dudi.coa**(**res, scannf **=** **FALSE**, nf **=** 2**)**

init_d **<-** scale**(**fit2**$**c1**[**,1**])[**,1**]**

sv**$**value**[**sv**$**parnum**[**sv**$**name**==**'delta'**]]** **<-** init_d

mod **<-** mirt**(**res,1,technical**=**list**(**theta_lim**=**c**(-**4,4**))**

,quadpts**=**50,pars**=**sv,c**(**rep**(**name,itemnum**))**,customItems**=**list**(**GGUM**=**ci**)** ,SE**=TRUE**,SE.type**=**"central",optimizer**=**"nlminb",control**=**list**(**rel.tol=1e-4,iter.max=10**)**,TOL**=**1e**-**4**)**

coef**(**mod, simplify **=** **TRUE)**

fscores(mod, method = "EAP",full.scores.SE=TRUE)

**R** syntax of **mirt** for estimating parameters of unidimensional graded unfolding model (GUM; Luo, 2001) for four-point Likert scale

GUM <- function(x,Theta,ncat){

xi_rho1 = cosh((0.5*(2*CC+1)+1-1)*x[2]*x[3])/cosh((0.5*(2*CC+1)-1)*x[2]*x[3])

sumTD = x[2]*(Theta-x[1])

xi_td1 = cosh((0.5*(2*CC+1)+1-1)*(sumTD))/cosh((0.5*(2*CC+1)-1)*(sumTD))

q1 = xi_td1/(xi_rho1 + xi_td1)

p1 = xi_rho1/(xi_rho1 + xi_td1)

xi_rho2 = cosh((0.5*(2*CC+1)+1-2)*x[2]*x[4])/cosh((0.5*(2*CC+1)-2)*x[2]*x[4])

xi_td2 = cosh((0.5*(2*CC+1)+1-2)*(sumTD))/cosh((0.5*(2*CC+1)-2)*(sumTD))

q2 = xi_td2/(xi_rho2 + xi_td2)

p2 = xi_rho2/(xi_rho2 + xi_td2)

xi_rho3 = cosh((0.5*(2*CC+1)+1-3)*x[2]*x[5])/cosh((0.5*(2*CC+1)-3)*x[2]*x[5])

xi_td3 = cosh((0.5*(2*CC+1)+1-3)*(sumTD))/cosh((0.5*(2*CC+1)-3)*(sumTD))

q3 = xi_td3/(xi_rho3 + xi_td3)

p3 = xi_rho3/(xi_rho3 + xi_td3)

nu1 = q1*q2*q3

nu2 = p1*q2*q3

nu3 = p1*p2*q3

nu4 = p1*p2*p3

de = nu1 + nu2 + nu3 + nu4

cbind(nu1/de, nu2/de, nu3/de, nu4/de)

}

res = read.table(file = "res.txt") # read your data (people × number of items)

itemnum = ncol(res)

library("mirt")

dim0 = 1 # unidimensionl scale

point = 4 # four-point scale

CC = point - 1

name = "Unfolding"

para = c(delta=0,alpha=1.5,rho1=1,rho2=1,rho3=1) # four point

toEst = rep(TRUE, dim0+dim0+CC)

lbound = c(rep(-Inf,dim0),rep(1e-5,dim0),rep(1e-5,CC))

ubound = c(rep(Inf,dim0),rep(Inf,dim0),rep(Inf,CC))

ci = createItem(name,par=para,est=toEst,P=GUM, lbound=lbound,ubound=ubound)

sv <- mirt(res, 1, c(rep(name,itemnum)), customItems=list(Unfolding=ci), pars='values')

# ESSENTIAL: set initial sign of delta for each item

# e.g., sv$value[1] = -1 and etc.

# equal threshold constraint

indrho1 = sv$parnum[sv$name == 'rho1']

indrho2 = sv$parnum[sv$name == 'rho2']

indrho3 = sv$parnum[sv$name == 'rho3']

rhoConstraint = list(c(indrho1),c(indrho2),c(indrho3))

# run mirt function

mod = mirt(res, 1, constrain = rhoConstraint, optimizer="nlminb", control=list(iter.max=20), pars=sv,method = 'EM',TOL=1e-4,SE=TRUE,SE.type="central", c(rep(name,itemnum)),technical= list(theta_lim=c(-4,4)),customItems=list(Unfolding=ci) )

coef(mod, printSE = TRUE, as.data.frame = TRUE)

**R** syntax of **mirt** for estimating parameters of three-dimensional graded unfolding model for four-point Likert scale

MGUM <- function(x,Theta,ncat){

C = ncat-1

xi_rho1 = cosh((0.5*(2*C+1)+1-1)*x[5])/cosh((0.5*(2*C+1)-1)*x[5])

sumTD = sqrt( (x[2]*(Theta[,1]-x[1]))^2 + (x[3]*(Theta[,2] - x[1]))^2 + (x[4]*(Theta[,3] - x[1]))^2 )

xi_td1 = cosh((0.5*(2*C+1)+1-1)*(sumTD))/cosh((0.5*(2*C+1)-1)*(sumTD))

q1 = xi_td1/(xi_rho1 + xi_td1)

p1 = xi_rho1/(xi_rho1 + xi_td1)

xi_rho2 = cosh((0.5*(2*C+1)+1-2)*x[6])/cosh((0.5*(2*C+1)-2)*x[6])

xi_td2 = cosh((0.5*(2*C+1)+1-2)*(sumTD))/cosh((0.5*(2*C+1)-2)*(sumTD))

q2 = xi_td2/(xi_rho2 + xi_td2)

p2 = xi_rho2/(xi_rho2 + xi_td2)

xi_rho3 = cosh((0.5*(2*C+1)+1-3)*x[7])/cosh((0.5*(2*C+1)-3)*x[7])

xi_td3 = cosh((0.5*(2*C+1)+1-3)*(sumTD))/cosh((0.5*(2*C+1)-3)*(sumTD))

q3 = xi_td3/(xi_rho3 + xi_td3)

p3 = xi_rho3/(xi_rho3 + xi_td3)

nu1 = q1*q2*q3

nu2 = p1*q2*q3

nu3 = p1*p2*q3

nu4 = p1*p2*p3

de = nu1 + nu2 + nu3 + nu4

prob = cbind(nu1/de, nu2/de, nu3/de, nu4/de)

prob

}

res = read.table(file = "res.txt") # read your data (people ×(itemnum*dim0))

dim0 = 3; point = 4; C = point-1

itemnum = 5 # the number of items for each dimension

itemnumTotal = itemnum*dim0

L = 1:itemnum

Lrec = matrix(NA,dim0,itemnum)

for (d in 1:dim0){ Lrec[d,] = L;L = L + itemnum}

library("mirt")

name = "Unfolding"

para = c(delta=0,alpha1=0.7,alpha2=0.5,alpha3=1.3,rho1=1,rho2=0.8,rho3=0.4)

toEst = rep(TRUE, 1+dim0+C)

lbound = c(rep(-Inf,1),rep(1e-5,dim0),rep(1e-5,C))

ci <- createItem(name,par=para,est=toEst,P=MGUM, lbound=lbound)

QQ <- matrix(0,itemnumTotal,dim0)

QQ[1:5,1] = 1; QQ[6:10,2] = 1; QQ[11:15,3] = 1; # Modify if needed

COV <- matrix(TRUE,dim0,dim0)

diag(COV) = FALSE

cmodel <- mirt.model(QQ, COV=COV)

sv <- mirt(res, cmodel, c(rep(name,itemnumTotal)), customItems=list(Unfolding=ci), pars='values')

# ESSENTIAL: Specify the sign of delta of each item

inddelta = sv$parnum[sv$name == 'delta']

SIGN = rep( c(-1,-1,-1,1,1) ,dim0)

sv$value[inddelta] = 1 * sign(SIGN)

# between-item design (i.e., one item only measures one dimension)

toFind = paste(c("alpha"), collapse = "");cmp = gregexpr(pattern =toFind, sv$name);index = sapply(cmp, "[[", 1) != -1

for (i in 1:itemnumTotal){

index2 = which(sv$item==paste("V",toString(i),sep="") & index)

index3 = index2[which(QQ[i,]==0)]

sv$value[index3] = 0

sv$est[index3] = FALSE

}

# which dimension in each row of sv

additemDim = numeric()

for (d in 1:dim0) additemDim = c(additemDim, rep(Lrec[d,],each=1+dim0+C) )

additemDim = c(additemDim, rep(0, dim0+ (dim0*dim0+dim0)/2 ) )

itemDim = additemDim

DIM = additemDim

for (d in 1:dim0){

for (i in 1:itemnum){

DIM[itemDim==Lrec[d,i]] = d

}

}

# equal threshold constraint

rhoConstraint = numeric()

for (d in 1:dim0){

indrho1 = sv$parnum[ DIM==d & sv$name == 'rho1' ]

indrho2 = sv$parnum[ DIM==d & sv$name == 'rho2' ]

indrho3 = sv$parnum[ DIM==d & sv$name == 'rho3' ]

rhoConstraint = append( rhoConstraint , list(c(indrho1),c(indrho2),c(indrho3)) )

}

# rhoConstraint = NULL # uncomment this if equal constraint is not needed

mod1 = mirt(res, cmodel, constrain = rhoConstraint, optimizer="nlminb", control=list(rel.tol=1e-10,abs.tol=1e-20,iter.max=20), pars=sv,method = EM,TOL=1e-03,SE=TRUE,SE.type="central", customItems=list(Unfolding=ci), c(rep(name,itemnumTotal)), technical=list(theta_lim=c(-4,4)),

quadpts=15)

coef(mod1, simplify=TRUE)
